# Immunogenicity, Safety, and Efficacy of a Tetravalent Dengue Vaccine in Children and Adolescents: An Analysis by Age Group

**DOI:** 10.1093/cid/ciae369

**Published:** 2024-07-12

**Authors:** Charissa Borja-Tabora, LakKumar Fernando, Eduardo Lopez Medina, Humberto Reynales, Luis Rivera, Xavier Saez-Llorens, Chukiat Sirivichayakul, Delia Yu, Nicolas Folschweiller, Kelley J Moss, Martina Rauscher, Vianney Tricou, Yuan Zhao, Shibadas Biswal

**Affiliations:** Clinical Research Division, Research Institute for Tropical Medicine, Muntinlupa, Philippines; Centre for Clinical Management of Dengue & Dengue Haemorrhagic Fever, Negombo General Hospital, Negombo, Sri Lanka; Centro de Estudios en Infectología Pediátrica CEIP, Universidad del Valle and Clínica Imbanaco Grupo Quironsalud, Cali, Colombia; Centro de Atención e Investigación Médica, CAIMED, Bogotá, Colombia; Hospital Maternidad Nuestra Senora de Altagracia, Santo Domingo, Dominican Republic; Hospital del Niño Dr. José Renán Esquivel, Sistema Nacional de Investigación at SENACYT, Centro de Vacunación Internacional (Cevaxin), Panama City, Panama; Department of Tropical Pediatrics, Faculty of Tropical Medicine, Mahidol University, Bangkok, Thailand; Pediatrics, De La Salle Medical and Health Sciences Institute, Dasmariñas, Philippines; Takeda Pharmaceuticals International AG, Zurich, Switzerland; Takeda Vaccines, Inc., Cambridge, Massachusetts, USA; Takeda Pharmaceuticals International AG, Zurich, Switzerland; Takeda Pharmaceuticals International AG, Zurich, Switzerland; Takeda Vaccines, Inc., Cambridge, Massachusetts, USA; Takeda Vaccines, Inc., Cambridge, Massachusetts, USA

**Keywords:** dengue vaccine, child, efficacy, immunogenicity, safety

## Abstract

**Background:**

Dengue is an increasing threat to global health. This exploratory analysis evaluated the immunogenicity, safety, and vaccine efficacy (VE) of a live-attenuated tetravalent dengue vaccine (TAK-003) in participants enrolled in the phase 3 DEN-301 trial (NCT02747927), stratified by baseline age (4–5 years, 6–11 years, or 12–16 years).

**Methods:**

Participants were randomized 2:1 to receive 2 doses of TAK-003, administered 3 months apart, or placebo. Dengue serostatus was evaluated at enrolment. VE against virologically confirmed dengue (VCD) and hospitalized VCD; immunogenicity (geometric mean titers [GMTs]); and safety were evaluated per age group through ∼4 years postvaccination.

**Results:**

VE against VCD across serotypes was 43.5% (95% confidence interval [CI]: 25.3%, 57.3%) for 4–5 year-olds; 63.5% (95% CI: 56.9%, 69.1%) for 6–11 year-olds, and 67.7% (95% CI: 57.8%, 75.2%) for 12–16 year-olds. VE against hospitalized VCD was 63.8% (95% CI: 21.1%, 83.4%), 85.1% (95% CI: 77.1%, 90.3%), and 89.7% (95% CI: 77.9%, 95.2%), for the 3 age groups, respectively. GMTs remained elevated against all 4 serotypes for ∼4 years postvaccination, with no evident differences across age groups. No clear differences in safety by age were identified.

**Conclusions:**

This exploratory analysis shows TAK-003 was efficacious in dengue prevention across age groups in children and adolescents 4–16 years of age living in dengue endemic areas. Relatively lower VE in 4–5 year-olds was potentially confounded by causative serotype distribution, small sample size, and VE by serotype, and should be considered in benefit-risk evaluations in this age group.

## Teaser

No major differences in immunogenicity, safety, or efficacy of tetravalent dengue vaccine (TAK-003) were evident by age group. These data support the use of TAK-003 in children and adolescents.

With the expanding range of mosquito vectors such as *Aedes aegypti*, into temperate and higher elevation regions [1, 2], together with urbanization resulting in higher population densities in suitable mosquito breeding habitats [3], dengue is a growing threat worldwide. Approximately half of the world's population currently live in areas where dengue is considered endemic, and in recent decades the number of cases has increased rapidly, from an estimated ∼500 000 reported to the World Health Organization (WHO) in 2000 up to 5.2 million in 2019 [4]. The spread of mosquito vectors into areas where populations have no previous exposure to infectious diseases such as dengue has the potential to cause explosive epidemics, which may present a high burden on healthcare services [4].

Current strategies for mitigating the impacts of dengue rely mostly on vector control and eradication [5–7]. A tetravalent vaccine (CYD-TDV) based on a yellow fever vaccine 17D backbone has been available since 2015 but is limited to individuals with evidence of previous dengue infection, due to increased risk of severe outcomes in dengue-naive recipients who later became infected with dengue [8, 9]. In 2022, a second tetravalent dengue vaccine (TAK-003) became available and is now approved in multiple countries worldwide without the need for serostatus testing prior to administration. TAK-003 has demonstrated immunogenicity and was well tolerated in phase 1 and 2 studies across children and adults living in dengue-endemic and non-endemic regions [10–14]. In the large-scale ongoing phase 3 efficacy study (DEN-301), which includes over 20 000 children and adolescents living in endemic regions of Asia and Latin America, vaccine efficacy (VE) against virologically confirmed dengue (VCD) was 80.2% (95% confidence interval [CI]: 73.3%, 85.3%) 1 year after vaccination, with cumulative VE against VCD and hospitalized VCD of 61.2% and 84.1% up to ∼4 years after receipt of the 2-dose vaccination series [15].

Although children, adolescents, and adults are all at risk of symptomatic or severe dengue, peak incidence varies across countries (eg, 4–9 year-olds in Colombia [16], 5–10 year-olds in India [17], 10–24 year-olds in Thailand [18], 15–24 year-olds in Brazil [19], 20–29 year-olds in the most recent severe outbreak in Sri Lanka [20], and 25–34 year-olds in Singapore [21]), with a higher disease burden in Asia compared with Latin America [22]. Developing vaccines, which induce robust immune responses, particularly for diseases with complex pathology such as dengue, can be particularly challenging in children whose immune systems are still developing [23]. In this analysis, we evaluated the immunogenicity, safety, and efficacy of TAK-003 in participants in the ongoing DEN-301 phase 3 study, stratified by age group at enrolment (4–5 years, 6–11 years, and 12–16 years).

## METHODS

### Study Design and Participants

Full details of study design, together with participant inclusion and exclusion criteria have been published previously [24–28]. In brief, eligible participants were randomized 2:1 to receive 2 doses of TAK-003 or placebo, administered 3 months apart (months 0 and 3). Randomization was stratified by region (Asia or Latin America) and by age group (4–5 years, 6–11 years, and 12–16 years) to ensure that each age range had a 2:1 ratio in each region (see Supplementary methods for further details). The study is registered at Clinicaltrials.gov: NCT02747927.

### Study Procedures

Participants (or their parents/guardians) were contacted at least weekly to monitor for febrile illness and potential dengue cases. Blood samples were taken from participants with febrile illnesses for dengue confirmation using serotype-specific reverse transcription polymerase chain reaction (RT-PCR). Hospitalization and management of VCD cases were according to standard local clinical practices, and investigators did not have real-time information available from the central laboratory during the study. Full details of study procedures have been published previously [24–28], and additional details are provided in the Supplementary materials.

### Outcomes

This pre-specified exploratory analysis evaluates the VE of TAK-003 in preventing VCD and hospitalized VCD from first dose to the end of Part 3 (approximately 57 months postvaccination) stratified by year of age and age group (4–5 years, 6–11 years, or 12–16 years) at enrolment. These age groups were selected as part of the study design, based on the differences in adverse event reporting in younger (<6 years) and older (≥6 years) participants. Immunogenicity was evaluated in terms of geometric mean titers (GMTs) of serotype-specific neutralizing antibodies and seropositivity rates. Solicited and unsolicited AEs, SAEs, AEs leading to study discontinuation, SAEs related to the investigational product, and deaths during the study are reported for each age group.

### Statistical Analysis

Full details of the sample size calculations for the primary and secondary objectives together with the statistical methodology have been published previously. All exploratory endpoints were reported descriptively, with no formal statistical analysis performed (see Supplementary materials). Analyses were performed using SAS version 9.4.

## RESULTS

In total, 20 099 participants were randomized and received either TAK-003 (n = 13 401) or placebo (n = 6698). The mean age in both treatment arms was 9.6 years, with 12.7% of participants aged 4–5 years, 55.2%–55.3% aged 6–11 years, and 32.1% aged 12–16 years (Table 1). Overall, 27.7% were seronegative at baseline, with fewer older participants being seronegative at baseline (16%) compared with the youngest age group (41%). The percentage of seronegative participants was balanced between the treatment groups for each age group. Overall, 94.2% of 4–5 year-olds, 93.9% of 6–11 year-olds, and 84.7% of 12–16 year-olds completed the study through the end of Part 3 (approximately 57 months postvaccination; median duration of follow-up after the second dose was 1641 days).

**Table 1.** Demographics and Baseline Characteristics (Safety Set)

Characteristic	Placebo n = 6687	TAK-003 n = 13 380
Mean age, y (SD)	9.6 (3.34)	9.6 (3.36)
Participants per age group		
4–5 y, n (%)	846 (12.7)	1702 (12.7)
6–11 y, n (%)	3697 (55.3)	7387 (55.2)
12–16 y, n (%)	2144 (32.1)	4291 (32.1)
Asia, n (%)	2993 (44.8)	5996 (44.8)
Latin America, n (%)	3694 (55.2)	7384 (55.2)
Seronegative, n (%)^a^	1832 (27.4)	3714 (27.8)
4–5 y, n (%)	355 (42.0)	696 (40.9)
6–11 y, n (%)	1128 (30.5)	2315 (31.4)
12–16 y, n (%)	349 (16.3)	703 (16.4)

^a^Baseline serostatus data were available for 6684 and 13 375 safety set participants in the placebo and TAK-003 groups, respectively.

Abbreviation: SD, standard deviation.

### VCD Causative Serotypes in Placebo Recipients

**Figure 1.**
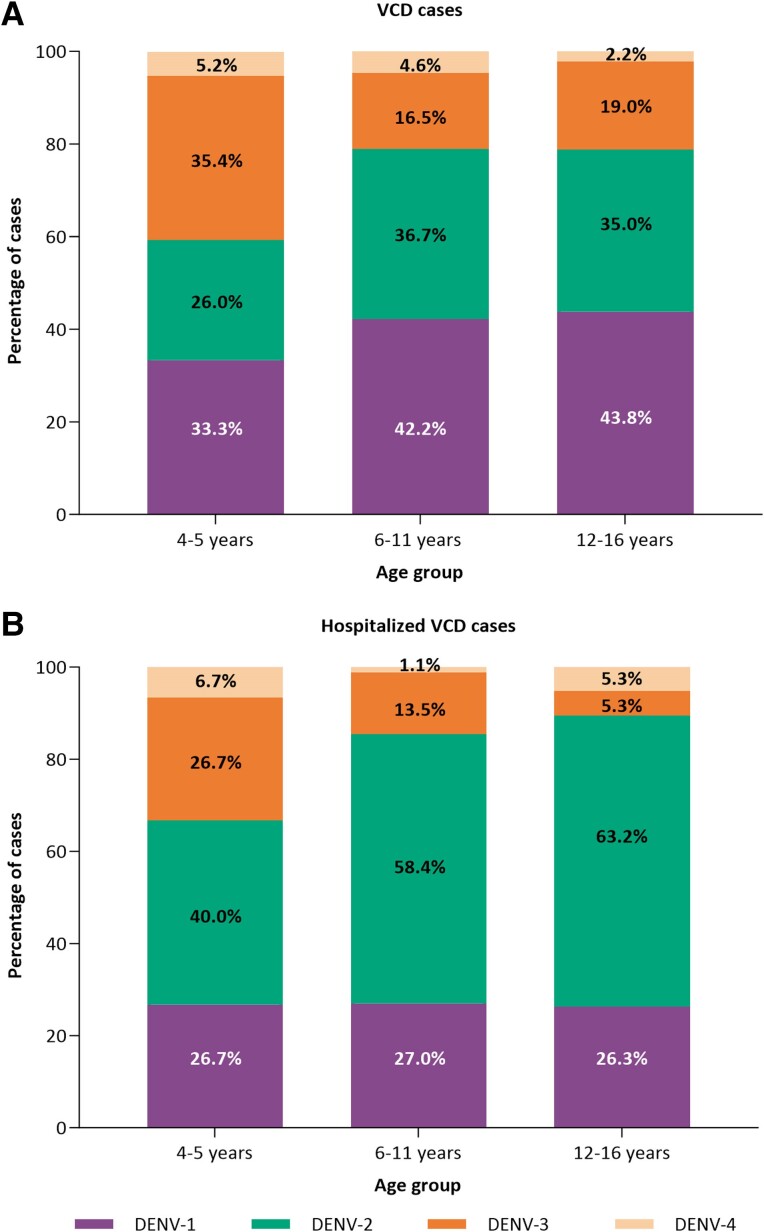
Causative serotype of (*A*) VCD cases and (*B*) hospitalized VCD cases in the placebo group by age group (safety set). Abbreviation: VCD, virologically confirmed dengue.

Across age groups, 560 cases of VCD were recorded from first vaccination to end of Part 3 in the placebo group, and 447 in the TAK-003 group. The predominant causative serotype in placebo recipients varied by age, with DENV-1 and DENV-3 causing the most cases in 4–5 year-olds (33.3% and 35.4%, respectively) compared with DENV-1 and DENV-2 in children aged ≥6 years (6–11 year-olds—DENV-1: 42.2%; DENV-2: 36.7%; 12–16 year-olds—DENV-1: 43.8%; DENV-2: 35.0%; Figure 1*A*). DENV-1 and DENV-2 caused the majority of hospitalized cases across age groups (66.7%–89.5%), whereas DENV-3 caused proportionally more hospitalized cases in 4–5 year-olds than in older children (Figure 1*B*).

### VE by Age Group and Serostatus

**Figure 2.**
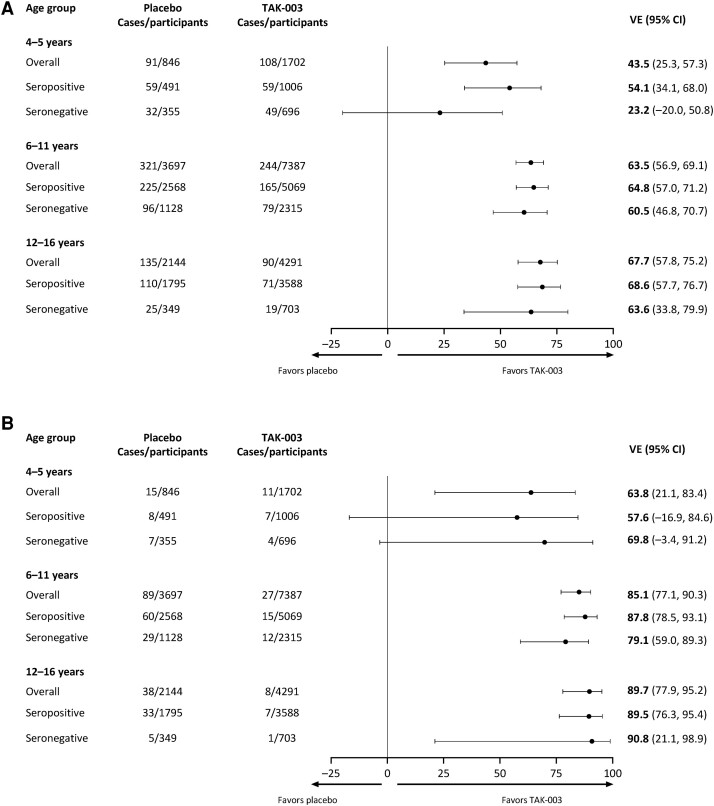
Efficacy against (*A*) VCD and (*B*) hospitalized VCD from the first vaccination to end of Part 3 by age group and baseline serostatus (safety set). Abbreviation: CI, confidence interval; VCD, virologically confirmed dengue.

Across serotypes and baseline serostatus, VE against VCD ranged from 43.5% (95% CI: 25.3%, 57.3%) in 4–5 year-olds through to 67.7% (95% CI: 57.8%, 75.2%) in 12–16 year-olds (Figure 2*A*). In participants ≥6 years of age, VE estimates were similar irrespective of baseline serostatus; in 6–11 year-olds VE was 64.8% (95% CI: 57.0%, 71.2%) in seropositive and 60.5% (95% CI: 46.8%, 70.7%) in seronegative participants; in 12–16 year-olds these were 68.6% (95% CI: 57.7%, 76.7%) and 63.6% (95% CI: 33.8%, 79.9%), respectively. VE in 4–5 year-olds was 54.1% (95% CI: 34.1%, 68.0%) in seropositive and 23.2% (95% CI: −20.0%, 50.8%) in seronegative participants.

TAK-003 also demonstrated efficacy across baseline serostatus against hospitalized VCD in all 3 age groups (Figure 2*B*). Across baseline serostatuses, VE against hospitalized VCD was 63.8% (95% CI: 21.1%, 83.4%) in the 4–5 year-olds, 85.1% (95% CI: 77.1%, 90.3%) in 6–11 year-olds, and 89.7% (95% CI: 77.9%, 95.2%) in 12–16 year-olds. VE estimates against individual serotypes are presented in Supplementary Table 1.

### VE by Year of Age

Estimates of VE of TAK-003 against VCD by year of age ranged from 49.1% (95% CI: 18.8%, 68.0%) in 9 year-olds to 76.9% (95% CI: 26.1%, 92.8%) in 16 year-olds in seropositive participants (Supplementary Table 2). More variation was seen in seronegative participants, ranging from −30.2% (−1152.1%, 86.5%) in 15 year-olds to 92.7% (95% CI: 67.4%, 98.3%) in 12 year-olds, with very few cases in children >12 years of age.

VE against hospitalized VCD was generally >75% across age groups in seropositive participants, with the exception of 4 year-olds (VE 3.1%, 95% CI: −287.4%, 75.8%) and 16 year-olds (68.0%, 95% CI: −252.9%, 97.1%). In seronegative participants, VE ranged from 51.3% to 100% in children aged 4–12 years, although was associated with large confidence intervals, and no cases reported in older children (Supplementary Table 3).

### VE by Year of Study and Serostatus

Evaluation of VE against VCD by individual study year showed substantial year-by-year variation in 4–5 year-olds and seronegative 12–16 year-olds (Supplementary Table 4). Waning efficacy was observed in 6–11 year-olds and seropositive 12–16 year-olds, falling from approximately 80% at the end of the first year postvaccination to 50%–60% by the end of Year 4. Year-by-year estimates of VE against hospitalized VCD were very variable for 4–5 year-olds, with very few hospitalized cases reported (Supplementary Table 5). Few cases were reported in 12–16 year-old seronegative participants. In contrast to VCD, no clear pattern of waning VE was observed against hospitalized VCD across the 3 age groups.

### Clinical Signs and Symptoms of VCD by Age Group and Baseline Serostatus

No clear differences in clinical signs or symptoms of cases were observed by baseline serostatus, age group, or treatment (Supplementary Tables 6–8). However, this analysis was also limited by small case counts in certain age groups.

In total, 3 severe dengue cases were reported in 4–5 year-olds (1 seronegative, 2 seropositive), 5 in 6–11 year-olds (1 seronegative, 4 seropositive), and 0 in 12–16 year-olds. In total, 4 cases of DHF were reported in 4–5 year-olds, 10 in 6–11 year-olds, and 10 in 12–16 year-olds, with the majority of cases in baseline seropositive placebo recipients (Supplementary Table 9). Two of the cases in the TAK-003 group (4–5 years seronegative and 4–5 years seropositive) and one in the placebo group (6–11 years seropositive) met both severe dengue and DHF criteria and were included in both categories.

### Immunogenicity

**Figure 3.**
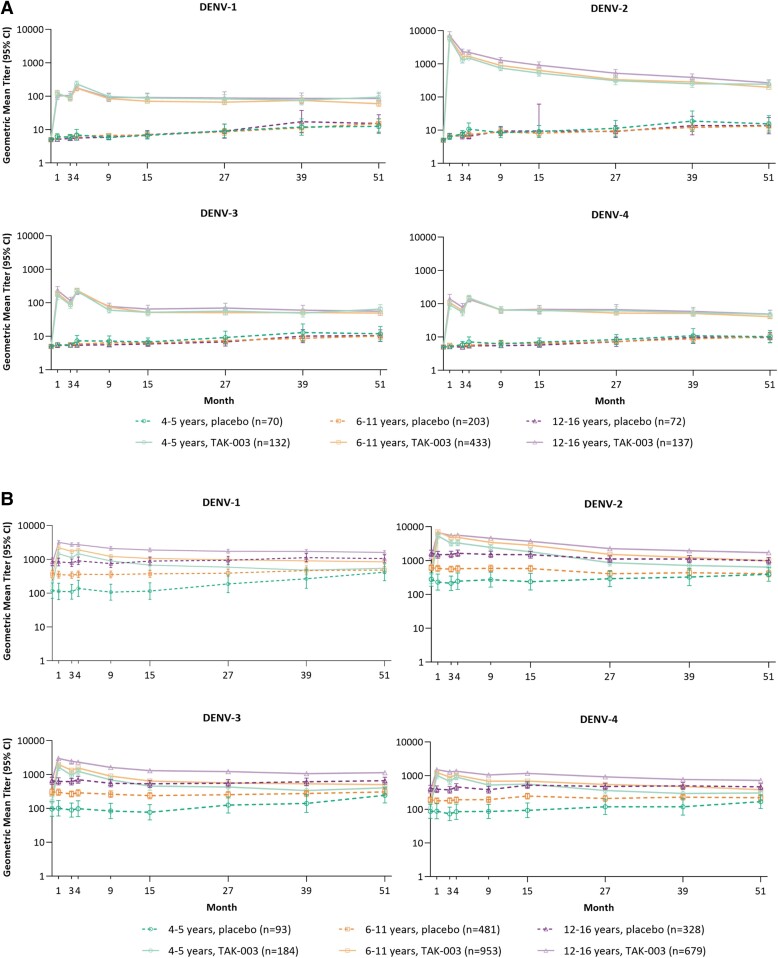
GMTs of neutralizing antibodies over time by age group in (*A*) baseline seronegative and (*B*) baseline seropositive participants (PPS immunogenicity subset). Note that not all participants may have had evaluable samples at each timepoint. Abbreviations: CI, confidence interval; GMT, geometric mean titer; PPS, per-protocol set.

In seronegative participants, no clear differences in GMTs postvaccination were evident by age group. While some antibody waning occurred, particularly against DENV-2, GMTs in the TAK-003 group were maintained above placebo levels throughout the study (Figure 3*A*).

In seropositive participants, GMTs were highest in the oldest age group, and lowest in the 4–5 year-olds, in both the placebo and TAK-003 groups (Figure 3*B*). Some waning occurred postvaccination in the TAK-003 group but GMTs remained above placebo levels within each age group throughout the analyzed time period. GMTs increased gradually in the placebo group over time in all age groups.

Rates of tetravalent seropositivity were high one month post-second dose and maintained through Month 51 in both baseline seronegative and seropositive participants. Rates were similar across age groups, with higher rates in baseline seropositive participants (Supplementary Figure 1).

### Safety

No clear differences were observed in rates of solicited local and systemic AEs or unsolicited AEs across age groups, as reported previously [26] (Supplementary Tables 10 and 11). Rates of AEs leading to discontinuation were low across all age groups (Supplementary Table 11). In Parts 1 and 2 of the study, rates of SAEs were similar across age groups and serostatus, with 2.6% to 6.8% of participants reporting SAEs. This trend was continued to Part 3 of the study, where 4.7% to 6.9% of participants reported SAEs across age groups (Table 2). The most commonly reported SAEs in all age groups were gastrointestinal disorders, infections and infestations, and injury, poisoning and procedural complications (Supplementary Tables 12 and 13) In total, 4 participants in the placebo group and 1 in the TAK-003 experienced SAEs considered related to the study interventions; these all occurred during Parts 1 and 2 of the study. Across all 3 study parts (randomization ratio 2:1 for TAK-003:placebo), SAEs leading to discontinuation were recorded for 1 participant in the 4–5 years group (placebo), 15 in the 6–11 years group (TAK-003: 11, placebo: 4) and 27 in the 12–16 years group (TAK-003: 18, placebo: 9). In total, 0 deaths occurred in 4–5 year-olds, 11 in 6–11 year-olds, and 12 in 12–16 years, none of which were considered related to the study vaccine.

**Table 2.** Number (%) of Participants Reporting SAEs, SAEs Considered Related to the Investigational Product, SAEs Leading to Discontinuation, and Deaths During Parts 1, 2, and 3 of the Study, by Age Group (Safety Set)

	Parts 1 and 2				Part 3			
	Seropositive		Seronegative		Seropositive		Seronegative	
	Placebo	TAK-003	Placebo	TAK-003	Placebo	TAK-003	Placebo	TAK-003
4–5 y	*n =* 491	*n =* 1006	*n =* 355	*n =* 696	*n =* 491	*n =* 1006	*n =* 355	*n =* 696
SAEs	18 (3.7)	42 (4.2)	19 (5.4)	47 (6.8)	28 (5.7)	44 (4.9)	18 (5.1)	40 (5.7)
Related SAEs	0	0	0	0	0	0	0	0
SAEs leading to discontinuation	1 (0.2)	0	0	0	0	0	0	0
Deaths	0	0	0	0	0	0	0	0
6–11 y	*n =* 2568	*n =* 5069	*n =* 1128	*n =* 2315	*n =* 2568	*n =* 5069	*n =* 1128	*n =* 2315
SAEs	139 (5.4)	210 (4.1)	56 (5.0)	86 (3.7)	146 (5.7)	239 (4.7)	63 (5.6)	108 (4.7)
Related SAEs	0	0	3 (0.3)	0	0	0	0	0
SAEs leading to discontinuation	1 (<0.1)	4 (<0.1)	1 (<0.1)	1 (<0.1)	2 (<0.1)	4 (<0.1)	0	2 (<0.1)
Deaths	1 (<0.1)	2 (<0.1)	0	0	2 (<0.1)	4 (<0.1)	0	2 (<0.1)
12–16 y	*n =* 1795	*n =* 3588	*n =* 349	*n =* 703	*n =* 1795	*n =* 3588	*n =* 349	*n =* 703
SAEs	83 (4.6)	132 (3.7)	9 (2.6)	21 (3.0)	117 (6.5)	198 (5.5)	24 (6.9)	35 (5.0)
Related SAEs	1 (<0.1)	1 (<0.1)	0	0	0	0	0	0
SAEs leading to discontinuation	4 (0.2)	12 (0.3)	1 (0.3)	1 (0.1)	3 (0.2)	5 (0.1)	1 (0.3)	0
Deaths	0	3 (<0.1)	0	0	3 (0.2)	5 (0.1)	1 (0.3)	0

Abbreviation: SAE, serious adverse event.

## DISCUSSION

This exploratory analysis shows similar trends in immunogenicity and no relevant differences in safety of TAK-003 by age group. Some waning of antibody titers was observed in all age groups through 51 months postvaccination, irrespective of baseline serostatus. In baseline seropositive participants, immunogenicity appeared higher in the older age group in both TAK-003 and placebo recipients, likely due to the higher proportion of participants who had been pre-exposed to multiple dengue serotypes compared with younger age groups. Although neutralizing antibody titers do not necessarily predict disease protection, VE estimates against VCD and hospitalized VCD were similar in 6–11 year-olds and 12–16 year-olds, irrespective of serostatus, but were lower in children aged 4–5 years.

Although lower VE estimates were noted in 4–5 year-olds, exploratory analysis was confounded by factors that precluded conclusion of an age effect such as small sample size, serotype distribution, and variable VE by serotype in seronegative participants. In total, the 4–5 years age group accounted for 12.7% of the total number of participants, and therefore estimates are likely less precise than for the other two age groups. Variable VE by serotype also played a role as the Philippines had the highest rates of enrolment of 4–5 year-olds and the majority of cases were attributable to DENV-3. In the overall study population, no efficacy against DENV-3 was noted in baseline seronegative participants [24–27]. Reports suggest that DENV-3 along with DENV-1 likely cause more symptomatic primary infections than the other 2 serotypes [29]. This, combined with the highest percentage of seronegative participants and smallest sample size in this age group, had the potential to confound the estimates, resulting in larger confidence intervals around VE estimates compared with the 2 older groups. Year-by-year estimates of VE against VCD in this age group showed fluctuations from 2.5% to 92.5%, with even greater variation against hospitalized VCD. Further granular analysis by individual age showed no clear monotonic trends in VE by individual year of age, therefore the lower VE associated with the 4–5 year age group should be interpreted with caution. Importantly, no increased risks were associated with use in the youngest age group, with safety profiles similar to those seen for older children. No clear age effects were seen in the assessment of immunogenicity data, and incidence rates of dengue were highest in the youngest age group, indicating that even with 43.5% efficacy against VCD and 63.8% against hospitalized VCD, TAK-003 would still result in a substantial absolute risk reduction; findings which should be taken into consideration in benefit-risk evaluations for use of TAK-003 in younger children.

The lack of efficacy against DENV-3 in seronegative participants has been discussed at length previously, together with the high proportion of DENV-3 hospitalizations in Sri Lanka (according to local practices) and lack of sufficient data for assessment of efficacy against DENV-4 in seronegative participants [27, 28]. Any potential risks associated with DENV-3 or DENV-4 infection, which could not be robustly evaluated by age group due to low case numbers, continue to be monitored closely as part of risk management plan, along with a post-licensure effectiveness study planned.

The data from this subgroup analysis support the indication for use of the TAK-003 in children and adolescents living in dengue endemic areas, without the need for prior serostatus testing. The Strategic Advisory Group of Experts (SAGE) on Immunization have recommended for programmatic use that TAK-003 be introduced for children aged 6–16 years in settings with high dengue disease burden and high transmission intensity, in order to maximize public health benefits. Introduction of vaccination is recommended approximately 1–2 years prior to the age-specific peak incidence in dengue-related hospitalizations, although it is not yet clear what the recommendations will be in situations where this peak age is younger than the recommended age range [30]. Currently, TAK-003 is licensed for use in adults and children with a lower age limit of 4 years in Argentina, Brazil, Colombia, Thailand, Vietnam, Israel, the European Union, European Economic Area, and the United Kingdom, and 6 years in Indonesia. Although evaluation of TAK-003 efficacy has not been performed in adults, owing to difficulties in potential enrolment of seronegative adults living in dengue-endemic regions, safety and immunogenicity studies performed in adults in both endemic and non-endemic regions [11, 13, 31–33], together with immunobridging data support its use in adults [10, 34]. Additionally, plans are under development to evaluate the use of TAK-003 in younger (<4 years of age) and older (≥60 years of age) individuals. It should, however, be noted that although currently available data do not conclude an age effect in TAK-003 profile, it is imperative that the vaccine is used according to the locally-approved indications and recommendations.

One of the strengths of this analysis was that it utilized long-term data collected from a large-scale ongoing clinical trial which included approximately 20 000 children and adolescents. Although the study was not designed specifically for analysis by age group, randomization was stratified so that the treatment groups were balanced for each of the three age groups, which increased the robustness of the current analysis. However, the analysis was not sufficiently powered for testing of statistical hypotheses, hence the results have been presented descriptively. Also, it should be noted that the last 18 months of the follow-up period coincided with the coronavirus disease 2019 (COVID-19) pandemic, which reduced the reported incidence of dengue and impacted healthcare responses [35, 36]. Despite these challenges, we feel that this analysis is robust enough to conclude that there were no substantial age-related differences in vaccine response or safety. Additionally, evaluation of a booster dose administered 4 years after primary vaccination is ongoing, and those data will be further explored for any potential age-affect.

In summary, in this analysis, TAK-003 demonstrated efficacy against both symptomatic and hospitalized dengue across all 3 age groups with similar immunogenicity and safety profiles. Lower VE associated with large confidence intervals was noted in children aged 4–5 years versus older children and should be considered in benefit-risk assessment of vaccination in this age group. Overall, the results of this evaluation provide evidence for the potential use of TAK-003 in children and adolescents living in areas considered endemic for dengue, irrespective of previous dengue infection history.

## Supplementary Data

Supplementary materials are available at *Clinical Infectious Diseases* online. Consisting of data provided by the authors to benefit the reader, the posted materials are not copyedited and are the sole responsibility of the authors, so questions or comments should be addressed to the corresponding author.

## Supplementary Material

ciae369_Supplementary_Data

## Notes

***Author contributions.*** C. B.-T., R. D., L. K. F., E. L. M., H. R., L. R., X. S.-L., C. S., and D. Y. were the investigators. S. B. designed the study. S. B., V. T., and N. F. managed the trial. N. F., M. R., V. T., Y. Z., and S. B. reviewed the data. N. F., K. J. M., M. R., V. T., Y. Z., and S. B. analyzed and interpreted the data. V. T. managed the publication. All authors had full access to the presented data, provided critical input during manuscript preparation, and read and approved the manuscript for submission.

***Acknowledgments.*** Medical writing and editorial support was provided by Jenny Engelmoer, PhD, of Sula Communications BV, and by Excel Medical Affairs, both funded by Takeda Vaccines, Inc.

The authors sincerely thank the trial participants and their parents/guardians who agreed to take part in the trial, the data monitoring committee members, the DCAC members, the Takeda expanded study team, the study team at PPD, and all the trial staff in each of the countries. The authors also acknowledge the contribution of the late Dan Stinchcomb, PhD, and Inviragen, Inc. (Fort Collins, Colorado, USA) to the initial developmental work of TAK-003 (Inviragen, Inc. was subsequently acquired by Takeda).

***Data sharing*.** The data sets, including the redacted study protocol, redacted statistical analysis plan, and individual participants' data supporting the results of the completed study, will be made available within 3 months from initial request, to researchers who provide a methodologically sound proposal. The data will be provided after its de-identification, in compliance with applicable privacy laws, data protection and requirements for consent and anonymization. Data requests should follow the process described in the Data Sharing section on https://clinicaltrials.takeda.com/ and https://vivli.org/ourmember/takeda/.

***Financial support.*** This work was funded by Takeda Vaccines, Inc., Cambridge, Massachusetts, USA.

## References

[1] Colón-González FJ, Sewe MO, Tompkins AM, et al Projecting the risk of mosquito-borne diseases in a warmer and more populated world: a multi-model, multi-scenario intercomparison modelling study. Lancet Planet Health 2021; 5:e404–14. 34245711 10.1016/S2542-5196(21)00132-7 PMC8280459

[2] Kulkarni MA, Duguay C, Ost K. Charting the evidence for climate change impacts on the global spread of malaria and dengue and adaptive responses: a scoping review of reviews. Glob Health 2022; 18:1. 10.1186/s12992-021-00793-2 PMC8725488 34980187

[3] Kolimenakis A, Heinz S, Wilson ML, et al The role of urbanisation in the spread of Aedes mosquitoes and the diseases they transmit: a systematic review. PLoS Negl Trop Dis 2021; 15:e0009631. 34499653 10.1371/journal.pntd.0009631 PMC8428665

[4] World Health Organization . Dengue and severe dengue. Available at: https://www.who.int/news-room/fact-sheets/detail/dengue-and-severe-dengue. Accessed 13 June 2024.

[5] Li M, Yang T, Bui M, et al Suppressing mosquito populations with precision guided sterile males. Nat Commun 2021; 12:5374. 34508072 10.1038/s41467-021-25421-w PMC8433431

[6] Utarini A, Indriani C, Ahmad RA, et al Efficacy of Wolbachia-infected mosquito deployments for the control of dengue. N Engl J Med 2021; 384:2177–86. 34107180 10.1056/NEJMoa2030243 PMC8103655

[7] Rather IA, Parray HA, Lone JB, et al Prevention and control strategies to counter dengue virus infection. Front Cell Infect Microbiol 2017; 7:336. 28791258 10.3389/fcimb.2017.00336 PMC5524668

[8] Dengue vaccine: WHO position paper, September 2018—recommendations. Vaccine 2019; 37:4848–9. 30424888 10.1016/j.vaccine.2018.09.063

[9] Sridhar S, Luedtke A, Langevin E, et al Effect of dengue serostatus on dengue vaccine safety and efficacy. N Engl J Med 2018; 379:327–40. 29897841 10.1056/NEJMoa1800820

[10] Patel SS, Rauscher M, Kudela M, Pang H. Clinical safety experience of TAK-003 for dengue fever: a new tetravalent live attenuated vaccine candidate. Clin Infect Dis 2023; 76:e1350–9. 35639602 10.1093/cid/ciac418 PMC9907483

[11] Sirivichayakul C, Barranco-Santana EA, Rivera IE, et al Long-term safety and immunogenicity of a tetravalent dengue vaccine candidate in children and adults: a randomized, placebo-controlled, phase 2 study. J Infect Dis 2022; 225:1513–20. 32658250 10.1093/infdis/jiaa406 PMC9071315

[12] Tricou V, Sáez-Llorens X, Yu D, et al Safety and immunogenicity of a tetravalent dengue vaccine in children aged 2–17 years: a randomised, placebo-controlled, phase 2 trial. Lancet 2020; 395:1434–43. 32197107 10.1016/S0140-6736(20)30556-0

[13] Turner M, Papadimitriou A, Winkle P, et al Immunogenicity and safety of lyophilized and liquid dengue tetravalent vaccine candidate formulations in healthy adults: a randomized, phase 2 clinical trial. Hum Vaccin Immunother 2020; 16:2456–64. 32119591 10.1080/21645515.2020.1727697 PMC7644226

[14] Biswal S, Mendez Galvan JF, Macias Parra M, et al Immunogenicity and safety of a tetravalent dengue vaccine in dengue-naïve adolescents in Mexico City. Rev Panam Salud Publica 2021; 45:e67. 34131423 10.26633/RPSP.2021.67 PMC8196333

[15] Tricou V, Yu D, Reynales H, et al Long-term efficacy and safety of Takeda's dengue vaccine (TAK 003): 4·5-year results from a phase 3, randomised, double-blind, placebo controlled trial. Lancet Glob Health 2024; 12:e257–270. 38245116 10.1016/S2214-109X(23)00522-3

[16] Ricardo-Rivera SM, Aldana-Carrasco LM, Lozada-Martinez ID, et al Mapping dengue in children in a Colombian Caribbean Region: clinical and epidemiological analysis of more than 3500 cases. Infez Med 2022; 30:602–9. 36482961 10.53854/liim-3004-16 PMC9715006

[17] Sinha B, Goyal N, Kumar M, et al Incidence of lab-confirmed dengue fever in a pediatric cohort in Delhi, India. PLoS Negl Trop Dis 2022; 16:e0010333. 35390000 10.1371/journal.pntd.0010333 PMC9017938

[18] Thisyakorn U, Saokaew S, Gallagher E, et al Epidemiology and costs of dengue in Thailand: a systematic literature review. PLoS Negl Trop Dis 2022; 16:e0010966. 36534668 10.1371/journal.pntd.0010966 PMC9810168

[19] Godói IP, Da Silva LVD, Sarker AR, et al Economic and epidemiological impact of dengue illness over 16 years from a public health system perspective in Brazil to inform future health policies including the adoption of a dengue vaccine. Expert Rev Vaccines 2018; 17:1123–33. 30417706 10.1080/14760584.2018.1546581

[20] Tissera HA, Jayamanne BDW, Raut R, et al Severe dengue epidemic, Sri Lanka, 2017. Emerg Infect Dis 2020; 26:682–91. 32186490 10.3201/eid2604.190435 PMC7101108

[21] Ministry of Health Singapore . Communicable diseases surveillance in Singapore 2019 & 2020, Chapter 3: vector-borne diseases. Available at: https://www.moh.gov.sg/resources-statistics/reports/communicable-diseases-surveillance-in-singapore-2019-2020. Accessed 3 May 2024.

[22] L'Azou M, Moureau A, Sarti E, et al Symptomatic dengue in children in 10 Asian and Latin American countries. N Engl J Med 2016; 374:1155–66. 27007959 10.1056/NEJMoa1503877

[23] Kloc M, Ghobrial RM, Kuchar E, Lewicki S, Kubiak JZ. Development of child immunity in the context of COVID-19 pandemic. Clin Immunol 2020; 217:108510. 32544611 10.1016/j.clim.2020.108510 PMC7293525

[24] Biswal S, Borja-Tabora C, Martinez Vargas L, et al Efficacy of a tetravalent dengue vaccine in healthy children aged 4–16 years: a randomised, placebo-controlled, phase 3 trial. Lancet 2020; 395:1423–33. 32197105 10.1016/S0140-6736(20)30414-1

[25] Biswal S, Reynales H, Saez-Llorens X, et al Efficacy of a tetravalent dengue vaccine in healthy children and adolescents. N Engl J Med 2019; 381:2009–19. 31693803 10.1056/NEJMoa1903869

[26] López-Medina E, Biswal S, Saez-Llorens X, et al Efficacy of a dengue vaccine candidate (TAK-003) in healthy children and adolescents 2 years after vaccination. J Infect Dis 2022; 225:1521–32. 33319249 10.1093/infdis/jiaa761 PMC9071282

[27] Rivera L, Biswal S, Sáez-Llorens X, et al Three-year efficacy and safety of Takeda's dengue vaccine candidate (TAK-003). Clin Infect Dis 2022; 75:107–17. 34606595 10.1093/cid/ciab864 PMC9402653

[28] Tricou V, Essink B, Ervin JE, et al Immunogenicity and safety of concomitant and sequential administration of yellow fever YF-17D vaccine and tetravalent dengue vaccine candidate TAK-003: a phase 3 randomized, controlled study. PLoS Negl Trop Dis 2023; 17:e0011124. 36888687 10.1371/journal.pntd.0011124 PMC9994689

[29] Clapham H, Cummings DA, Nisalak A, et al Epidemiology of infant dengue cases illuminates serotype-specificity in the interaction between immunity and disease, and changes in transmission dynamics. PLoS Negl Trop Dis. 2015; 9:e0004262. 26658730 10.1371/journal.pntd.0004262 PMC4684242

[30] World Health Organization . Highlights from the meeting of the strategic advisory group of experts (SAGE) on immunization 25–29 September 2023. Available at: https://www.who.int/news-room/events/detail/2023/09/25/default-calendar/sage_meeting_september_2023. Accessed 12 October 2023.

[31] Tricou V, Low JG, Oh HM, et al Safety and immunogenicity of a single dose of a tetravalent dengue vaccine with two different serotype-2 potencies in adults in Singapore: a phase 2, double-blind, randomised, controlled trial. Vaccine 2020; 38:1513–9. 31843269 10.1016/j.vaccine.2019.11.061

[32] Patel SS, Winkle P, Faccin A, Nordio F, LeFevre I, Tsoukas CG. An open-label, phase 3 trial of TAK-003, a live attenuated dengue tetravalent vaccine, in healthy US adults: immunogenicity and safety when administered during the second half of a 24-month shelf-life. Hum Vaccin Immunother 2023; 19:2254964. 37846724 10.1080/21645515.2023.2254964 PMC10583633

[33] Tricou V, Winkle PJ, Tharenos LM, et al Consistency of immunogenicity in three consecutive lots of a tetravalent dengue vaccine candidate (TAK-003): a randomized placebo-controlled trial in US adults. Vaccine 2023; 41:6999–7006. 37884415 10.1016/j.vaccine.2023.09.049

[34] LeFevre I, Bravo L, Folschweiller N, et al Bridging the immunogenicity of a tetravalent dengue vaccine (TAK-003) from children and adolescents to adults. NPJ Vaccines 2023; 8:75. 37230978 10.1038/s41541-023-00670-6 PMC10208910

[35] Chen Y, Li N, Lourenço J, et al Measuring the effects of COVID-19-related disruption on dengue transmission in Southeast Asia and Latin America: a statistical modelling study. Lancet Infect Dis 2022; 22:657–67. 35247320 10.1016/S1473-3099(22)00025-1 PMC8890758

[36] Tangsathapornpong A, Thisyakorn U. Dengue amid COVID-19 pandemic. PLoS Glob Public Health 2023; 3:e0001558. 36962879 10.1371/journal.pgph.0001558 PMC10021186

