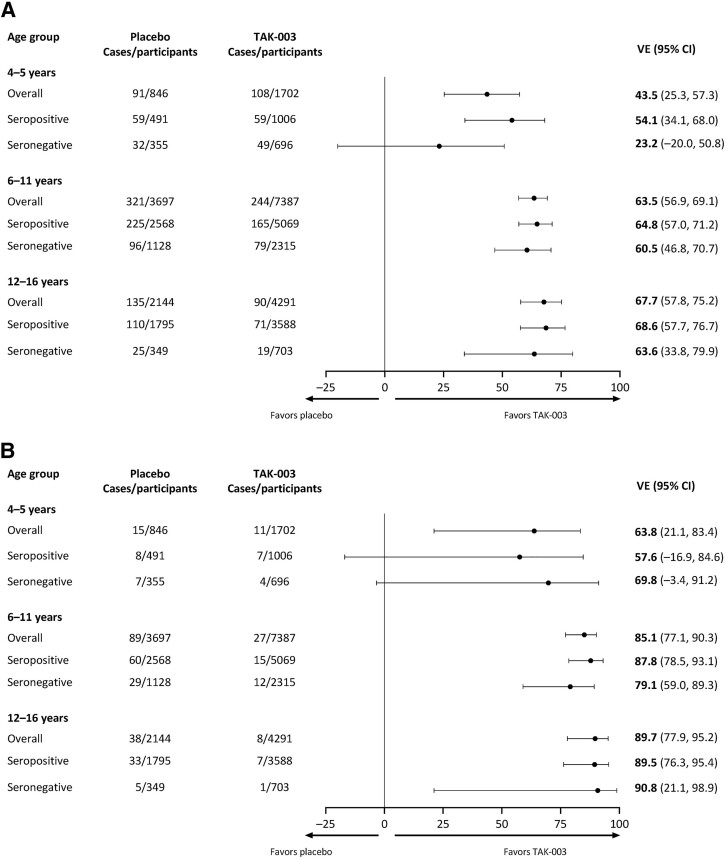# Correction to: Immunogenicity, Safety, and Efficacy of a Tetravalent Dengue Vaccine in Children and Adolescents: An Analysis by Age Group

**DOI:** 10.1093/cid/ciae504

**Published:** 2024-11-15

An error appeared in the corrected proof publication of this article (Borja-Tabora et al. “Immunogenicity, Safety, and Efficacy of a Tetravalent Dengue Vaccine in Children and Adolescents: An Analysis by Age Group.” *Clin Infect Dis*. https://doi.org/10.1093/cid/ciae369). For the 6-11 and 12-16 age groups in both parts of the figure, both rows read “seropositive,” whereas the bottom line of each group should read “seronegative.” A corrected version of the figure appears below.

**Figure:**