# Supplementary material for: Immunogenicity, Safety, and Efficacy of a Tetravalent Dengue Vaccine in Children and Adolescents: An Analysis by Age Group
Source: Clin Infect Dis. 2024 Jul 12;80(1):199–206. doi: 10.1093/cid/ciae369 (PMC11797386; doi:10.1093/cid/ciae369)

**Supplementary methods**

*Study design and participants*

The phase 3, double-blind, placebo-controlled randomized DEN-301 clinical trial is being conducted in healthy 4–16 year-olds living in eight countries considered endemic for dengue (Brazil, Colombia, the Dominican Republic, Nicaragua, Panama, the Philippines, Sri Lanka, and Thailand). Enrolment commenced in April 2016, with the first vaccine dose administered between September 2016 and March 2017. The study is being conducted in accordance with the Declaration of Helsinki and the principles of Good Clinical Practice, with applicable local regulations. Written informed assent/consent was obtained from participants and/or their parents or legal guardians prior to enrolment in the study. The protocol, its amendments, and informed consent and assent forms were approved by local ethics committees or institutional review boards, as appropriate, prior to commencement of the study. The study is registered at clinicaltrials.gov: NCT02747927. The study initially consisted of 3 parts (see [26]), but was extended following a protocol amendment to include an additional two-part booster phase, with participants aged 4–11 years at study entry eligible for a booster dose approximately 4 years after receipt of the second dose. The booster phase is currently ongoing, therefore this analysis contains data up to the end of Part 3 of the study, approximately 4.5 years after receipt of the primary vaccine series.

*Study procedures*

Pre-vaccination blood samples were taken on Day 1 to determine baseline dengue serostatus, and post-vaccination samples were taken one month after the second dose (Month 4) for evaluation of dengue-specific neutralizing antibodies, evaluated by microneutralization (MNT) assay. Baseline seropositivity was defined as an MNT titer of ≥10 against at least one DENV serotype; seronegative was defined as an MNT titer <10 against all four DENV serotypes. A subgroup of 4000 participants (immunogenicity subset) was randomly selected for additional safety and immunogenicity assessments, with additional blood samples taken at Months 1, 3, 9, 15, and then annually (see [26] for details). In the subgroup, local and systemic solicited adverse events (AEs) were collected in subject diaries for 7 and 14 days, respectively, following each vaccine dose; unsolicited AEs were collected for 28 days after each vaccination. Serious AEs (SAEs) and AEs leading to discontinuation are being recorded for all study participants throughout the study.

*Statistical analysis*

Sample size calculations were not powered for the current exploratory analysis. VE, expressed as a percentage, was calculated by age group as 1−(λV / λC), where λV and λC denote the hazard rates for the TAK-003 and placebo groups, respectively. Hazard ratios and associated 95% confidence intervals were calculated per age group using a Cox proportional model. Immunogenicity endpoints are summarized using descriptive statistics and 95% CIs by group and for each visit; safety data are presented as the number (%) of participants reporting events.

**Supplementary Table 1.** Efficacy against VCD and hospitalized VCD from the first vaccination to end of Part 3 by age group, baseline serostatus, and DENV serotype (safety set)

Table

| **Age group** | **Serotype** | **VCD Cases Placebo**  **n/N (%)** | **VCD Cases**  **TAK-003**  **n/N (%)** | **VE against VCD (95% CI)** | **Hospitalized VCD Cases Placebo**  **n/N (%)** | **Hospitalized VCD Cases**  **TAK-003**  **n/N (%)** | **VE against hospitalized VCD (95% CI)** |
| --- | --- | --- | --- | --- | --- | --- | --- |
| *Seropositive* |  |  |  |  |  |  |  |
| 4-5 years | DENV-1 | 21/491 (4.3) | 25/1006 (2.5) | 43.2 (-1.5, 68.2) | 2/491 (0.4) | 4/1006 (0.4) | 3.1 (-429.1, 82.3) |
|  | DENV-2 | 13/491 (2.6) | 7/1006 (0.7) | 74.2 (35.5, 89.7) | 4/491 (0.8) | 0/1006 (0) | 100.0 (NE) |
|  | DENV-3 | 26/491 (5.3) | 24/1006 (2.4) | 56.8 (24.8, 75.2) | 2/491 (0.4) | 3/1006 (0.3) | 26.8 (-338.3, 87.8) |
|  | DENV-4 | 3/491 (0.6) | 3/1006 (0.3) | 51.4 (-140.7, 90.2) | 0/491 (0) | 0/1006 (0) | NE |
| 6-11 years | DENV-1 | 88/2568 (3.4) | 92/5069 (1.8) | 47.3 (29.4, 60.7) | 15/2568 (0.6) | 11/5069 (0.2) | 63.3 (20.1, 83.1) |
|  | DENV-2 | 80/2568 (3.1) | 27/5069 (0.5) | 83.4 (74.4, 89.3) | 32/2568 (1.2) | 2/5069 (<0.1) | 96.9 (87.2, 99.3) |
|  | DENV-3 | 47/2568 (1.8) | 41/5069 (0.8) | 58.1 (36.2, 72.4) | 12/2568 (0.5) | 2/5069 (<0.1) | 92.0 (64.1, 98.2) |
|  | DENV-4 | 14/2568 (0.5) | 5/5069 (<0.1) | 82.7 (51.9, 93.8) | 1/2568 (<0.1) | 0/5069 (0) | 100.0 (NE) |
| 12-16 years | DENV-1 | 42/1795 (2.3) | 16/3588 (0.4) | 81.1 (66.4, 89.4) | 7/1795 (0.4) | 1/3588 (<0.1) | 92.8 (41.9, 99.1) |
|  | DENV-2 | 42/1795 (2.3) | 20/3588 (0.6) | 76.5 (59.9, 86.2) | 23/1795 (1.3) | 3/3588 (<0.1) | 93.5 (78.4, 98.1) |
|  | DENV-3 | 24/1795 (1.3) | 31/3588 (0.9) | 35.6 (-9.8, 62.2) | 1/1795 (<0.1) | 3/3588 (<0.1) | -47.0 (-1314.1, 84.7) |
|  | DENV-4 | 3/1795 (0.2) | 4/3588 (0.1) | 33.4 (-197.4, 85.1) | 2/1795 (0.1) | 0/3588 (0) | 100.0 (NE) |
| *Seronegative* |  |  |  |  |  |  |  |
| 4-5 years | DENV-1 | 11/355 (3.1) | 20/696 (2.9) | 7.9 (-92.5, 55.9) | 2/355 (0.6) | 1/696 (0.1) | 74.1 (-186.6, 97.7) |
|  | DENV-2 | 12/355 (3.4) | 6/696 (0.9) | 75.5 (34.7, 90.8) | 2/355 (0.6) | 0/696 (0) | 100.0 (NE) |
|  | DENV-3 | 8/355 (2.3) | 18/696 (2.6) | -19.0 (-173.9, 48.3) | 2/355 (0.6) | 3/696 (0.4) | 16.9 (-397.2, 86.1) |
|  | DENV-4 | 2/355 (0.6) | 5/696 (0.7) | -20.8 (-522.9, 76.6) | 1/355 (0.3) | 0/696 (0) | 100.0 (NE) |
| 6-11 years | DENV-1 | 50/1128 (4.4) | 52/2315 (2.2) | 50.3 (26.7, 66.3) | 9/1128 (0.8) | 5/2315 (0.2) | 71.8 (15.9, 90.6) |
|  | DENV-2 | 40/1128 (3.5) | 8/2315 (0.3) | 90.1 (78.8, 95.4) | 20/1128 (1.8) | 0/2315 (0) | 100.0 (NE) |
|  | DENV-3 | 7/1128 (0.6) | 17/2315 (0.7) | -29.8 (-213.4, 46.2) | 0/1128 (0) | 7/2315 (0.3) | NE |
|  | DENV-4 | 1/1128 (<0.1) | 6/2315 (0.3) | -212.2 (-2493.2, 62.4) | 0/1128 (0) | 0/2315 (0) | NE (NE) |
| 12-16 years | DENV-1 | 18/349 (5.2) | 17/703 (2.4) | 53.9 (10.5, 76.2) | 3/349 (0.9) | 0/703 (0) | 100.0 (NE) |
|  | DENV-2 | 6/349 (1.7) | 0/703 (0) | 100.0 (NE) | 1/349 (0.3) | 0/703 (0) | 100.0 (NE) |
|  | DENV-3 | 1/349 (0.3) | 1/703 (0.1) | 54.5 (-627.4, 97.2) | 1/349 (0.3) | 1/703 (0.1) | 54.5 (-627.4, 97.2) |
|  | DENV-4 | 0/349 (0) | 1/703 (0.1) | <-999.99 (NE) | 0/349 (0) | 0/703 (0) | NE (NE) |

CI, confidence interval; NE, not evaluable; VCD, virologically confirmed dengue; VE, vaccine efficacy

n/N for cases represents number of virologically confirmed (hospitalized) dengue cases/number of participants evaluated

**Supplementary Table 2**. Efficacy of TAK-003 against VCD from first vaccination to the end of Part 3 by year of age and baseline serostatus (safety set)

| **Age** | **Baseline Seropositive** | | | **Baseline Seronegative** | | |
| --- | --- | --- | --- | --- | --- | --- |
|  | **Placebo**  n/N (%) | **TAK-003**  n/N (%) | **Vaccine Efficacy** % (95% CI) | **Placebo**  n/N (%) | **TAK-003**  n/N (%) | **Vaccine Efficacy**  % (95% CI) |
| **4 years** | 25/214 (11.7) | 27/428 (6.3) | 50.9 (15.4, 71.5) | 14/141 (9.9) | 23/313 (7.3) | 24.9 (-45.9, 61.4) |
| **5 years** | 34/277 (12.3) | 32/578 (5.5) | 56.6 (29.7, 73.2) | 18/214 (8.4) | 26/383 (6.8) | 21.3 (-43.6, 56.8) |
| **6 years** | 44/392 (11.2) | 31/794 (3.9) | 68.5 (50.1, 80.1) | 19/250 (7.6) | 18/488 (3.7) | 51.4 (7.3, 74.5) |
| **7 years** | 40/405 (9.9) | 23/811 (2.8) | 73.2 (55.3, 84.0) | 17/187 (9.1) | 14/434 (3.2) | 65.9 (30.8, 83.2) |
| **8 years** | 35/458 (7.6) | 32/877 (3.6) | 52.7 (23.6, 70.7) | 20/210 (9.5) | 20/433 (4.6) | 52.7 (12.0, 74.6) |
| **9 years** | 34/416 (8.2) | 37/849 (4.4) | 49.1 (18.8, 68.0) | 13/173 (7.5) | 10/372 (2.7) | 65.7 (21.7, 85.0) |
| **10 years** | 41/461 (8.9) | 19/791 (2.4) | 74.3 (55.8, 85.1) | 11/164 (6.7) | 5/323 (1.5) | 77.8 (36.2, 92.3) |
| **11 years** | 31/436 (7.1) | 23/947 (2.4) | 67.6 (44.5, 81.1) | 16/144 (11.1) | 12/265 (4.5) | 57.4 (9.4, 80.0) |
| **12 years** | 33/469 (7.0) | 21/957 (2.2) | 70.0 (48.2, 82.7) | 13/110 (11.8) | 2/212 (0.9) | 92.7 (67.4, 98.3) |
| **13 years** | 32/456 (7.0) | 19/867 (2.2) | 69.2 (45.7, 82.6) | 7/93 (7.5) | 8/195 (4.1) | 47.5 (-44.7, 81.0) |
| **14 years** | 22/385 (5.7) | 17/749 (2.3) | 61.1 (26.8, 79.4) | 3/73 (4.1) | 4/138 (2.9) | 29.0 (-219.3, 84.2) |
| **15 years** | 13/276 (4.7) | 10/619 (1.6) | 68.2 (27.6, 86.1) | 1/43 (2.3) | 3/92 (3.3) | -30.2 (-1152.1, 86.5) |
| **16 years** | 10/209 (4.8) | 4/396 (1.0) | 76.9 (26.1, 92.8) | 1/30 (3.3) | 2/66 (3.0) | 36.6 (-599.3, 94.3) |

CI, confidence interval

**Supplementary Table 3**. Efficacy of TAK-003 against hospitalized VCD from first vaccination to the end of Part 3 by year of age and baseline serostatus (safety set)

| **Age** | **Baseline Seropositive** | | | **Baseline Seronegative** | | |
| --- | --- | --- | --- | --- | --- | --- |
|  | **Placebo**  n/N (%) | **TAK-003**  n/N (%) | **Vaccine Efficacy** % (95% CI) | **Placebo**  n/N (%) | **TAK-003**  n/N (%) | **Vaccine Efficacy**  % (95% CI) |
| **4 years** | 3/214 (1.4) | 6/428 (1.4) | 3.1 (-287.4, 75.8) | 2/141 (1.4) | 0/313 (0.0) | 100 (NE, NE) |
| **5 years** | 5/277 (1.8) | 1/578 (0.2) | 90.6 (19.2, 98.9) | 5/214 (2.3) | 4/383 (1.0) | 56.3 (-62.6, 88.3) |
| **6 years** | 9/392 (2.3) | 2/794 (0.3) | 89.9 (53.3, 97.8) | 3/250 (1.2) | 1/488 (0.2) | 82.3 (-70.4, 98.2) |
| **7 years** | 8/405 (2.0) | 2/811 (0.2) | 88.1 (44.2, 97.5) | 5/187 (2.7) | 3/434 (0.7) | 74.7 (-6.0, 93.9) |
| **8 years** | 11/458 (2.4) | 4/877 (0.5) | 80.7 (39.3, 93.8) | 8/210 (3.8) | 4/433 (0.9) | 74.6 (15.6, 92.3) |
| **9 years** | 12/416 (2.9) | 3/849 (0.4) | 88.3 (58.5, 96.7) | 5/173 (2.9) | 1/372 (0.3) | 90.8 (21.7, 98.9) |
| **10 years** | 9/461 (2.0) | 3/791 (0.4) | 81.5 (31.6, 95.0) | 3/164 (1.8) | 3/323 (0.9) | 51.3 (-141.2, 90.2) |
| **11 years** | 11/436 (2.5) | 1/947 (0.1) | 96.0 (68.8, 99.5) | 5/144 (3.5) | 0/265 (0.0) | 100.0 (NE, NE) |
| **12 years** | 8/469 (1.7) | 4/957 (0.4) | 76.3 (21.2, 92.9) | 5/110 (4.5) | 1/212 (0.5) | 90.8 (21.1, 98.9) |
| **13 years** | 12/456 (2.6) | 2/867 (0.2) | 91.1 (60.2, 98.0) | 0/93 (0.0) | 0/195 (0.0) | NE (NE, NE) |
| **14 years** | 5/385 (1.3) | 0/749 (0.0) | 100.0 (NE, NE) | 0/73 (0.0) | 0/138 (0.0) | NE (NE, NE) |
| **15 years** | 6/276 (2.2) | 0/619 (0.0) | 100.0 (NE, NE) | 0/43 (0.0) | 0/92 (0.0) | NE (NE, NE) |
| **16 years** | 2/209 (1.0) | 1/396 (0.3) | 68.0 (-252.9, 97.1) | 0/30 (0.0) | 0/66 (0.0) | NE (NE, NE) |

CI, confidence interval; NE, not evaluable

**Supplementary Table 4.** Case counts of VCD and efficacy of TAK-003 for individual study years from 30 days after the second dose by age group and serostatus (per protocol set)

|  | **Year 1*** | **Year 2** | **Year 3** | **Year 4** | **Last 18 months**^†^ |
| --- | --- | --- | --- | --- | --- |
| **4-5 years** |  |  |  |  |  |
| **Seropositive (placebo/TAK-003)** | *n=464/957* | *n=461/948* | *n=457/941* | *n=454/936* | *n=454/936* |
| Case counts (placebo/TAK-003) | 18/7 | 13/15 | 16/34 | 6/1 | 6/1 |
| VE (95% CI) | 81.8 (56.4, 92.4) | 46.4 (–12.7, 74.5) | 2.5 (–76.6, 46.2) | 92.5 (37.9, 99.1) | 92.5 (37.9, 99.1) |
| **Seronegative (placebo/TAK-003)** | *n=337/662* | *n=334/655* | *n=331/652* | *n=327/644* | *n=327/644* |
| Case counts (placebo/TAK-003) | 5/6 | 6/15 | 16/17 | 2/2 | 3/8 |
| VE (95% CI) | 39.1 (–99.8, 81.4) | –23.7 (–219.1, 52.0) | 47.4 (–4.3, 73.4) | 51.2 (–247.1, 93.2) | –27.9 (–382.6, 66.1) |
| **6–11 years** |  |  |  |  |  |
| **Seropositive (placebo/TAK-003)** | *n=2423/4806* | *n=2415/4775* | *n=2400/4743* | *n=2381/4705* | *n=2381/4705* |
| Case counts (placebo/TAK-003) | 58/22 | 48/39 | 76/75 | 16/14 | 21/19 |
| VE (95% CI) | 82.0 (70.6, 89.0) | 60.8 (40.2, 74.3) | 52.6 (34.7, 65.5) | 58.7 (15.4, 79.8) | 57.5 (20.9, 77.1) |
| **Seronegative (placebo/TAK-003)** | *n=1065/2200* | *n=1060/2184* | *n=1051/2165* | *n=1045/2133* | *n=1044/2132* |
| Case counts (placebo/TAK-003) | 27/12 | 19/16 | 27/40 | 8/7 | 9/9 |
| VE (95% CI) | 78.4 (57.3, 89.1) | 59.7 (21.6, 79.3) | 30.0 (–14.0, 57.1) | 58.6 (–14.1, 85.0) | 52.5 (–19.7, 81.2) |
| **12–16 years** |  |  |  |  |  |
| **Seropositive (placebo/TAK-003)** | *n=1700/3402* | *n=1676/3355* | *n=1644/3284* | *n=1590/3179* | *n=1590/3179* |
| Case counts (placebo/TAK-003) | 34/12 | 16/10 | 37/30 | 7/7 | 8/10 |
| VE (95% CI) | 82.7 (66.5, 91.0) | 69.9 (33.7, 86.3) | 61.1 (37.1, 76.0) | 51.8 (–37.4, 83.1) | 40.2 (–51.5, 76.4) |
| **Seronegative (placebo/TAK-003)** | *n=324/669* | *n=321/659* | *n=316/648* | *n=305/626* | *n=305/626* |
| Case counts (placebo/TAK-003) | 7/2 | 4/2 | 8/12 | 3/2 | 5/2 |
| VE (95% CI) | 86.2 (33.8, 97.1) | 75.9 (–31.6, 95.6) | 29.2 (–73.2, 71.1) | 67.5 (–95.1, 94.6) | 80.9 (1.4, 96.3) |

Number of subjects evaluated each year may vary. Repeat episodes of VCD excluded from efficacy analysis.

Seronegative at baseline: seronegative to all four dengue serotypes. Seropositive at baseline: reciprocal neutralizing titer ≥10 for one or more dengue serotypes.
*30 days after 2^nd^ dose to end of 1^st^ year after 2^nd^ dose;

^†^This period generally includes Year 4 and the last 0.5 years were not uniform for all subjects.

CI, confidence interval; VCD, virologically confirmed dengue; VE, vaccine efficacy

**Supplementary Table 5.** Case counts of hospitalized VCD and efficacy of TAK-003 for individual study years from 30 days after the second dose by age group and serostatus (per protocol set)

|  | **Year 1*** | **Year 2** | **Year 3** | **Year 4** | **Last 18 months**^†^ |
| --- | --- | --- | --- | --- | --- |
| **4-5 years** |  |  |  |  |  |
| **Seropositive** | *n=464/957* | *n=461/948* | *n=457/941* | *n=454/936* | *n=454/936* |
| Case counts (placebo/TAK-003) | 1/1 | 3/2 | 1/4 | 2/0 | 2/0 |
| VE (95% CI) | 51.9 (–668.3, 97.0) | 68.7 (–87.1, 94.8) | –80.7 (–1517.3, 79.8) | 100 (NE, NE) | 100 (NE, NE) |
| **Seronegative** | *n=337/662* | *n=334/655* | *n=331/652* | *n=327/644* | *n=327/644* |
| Case counts (placebo/TAK-003) | 2/0 | 1/3 | 2/1 | 1/0 | 1/0 |
| VE (95% CI) | 100 (NE, NE) | –54.6 (–1387.6, 83.9) | 73.0 (–197.4, 97.6) | 100 (NE, NE) | 100 (NE, NE) |
| **6–11 years** |  |  |  |  |  |
| **Seropositive** | *n=2423/4806* | *n=2415/4775* | *n=2400/4743* | *n=2381/4705* | *n=2381/4705* |
| Case counts (placebo/TAK-003) | 19/1 | 11/2 | 18/5 | 5/1 | 7/2 |
| VE (95% CI) | 97.5 (81.4, 99.7) | 91.4 (61.0, 98.1) | 87.0 (65.0, 95.2) | 90.7 (20.6, 98.9) | 86.5 (34.9, 97.2) |
| **Seronegative** | *n=1065/2200* | *n=1060/2184* | *n=1051/2165* | *n=1045/2133* | *n=1044/2132* |
| Case counts (placebo/TAK-003) | 14/1 | 4/3 | 5/7 | 3/0 | 3/0 |
| VE (95% CI) | 96.4 (72.5, 99.5) | 64.4 (–59.0, 92.0) | 28.7 (–124.9, 77.4) | 100 (NE, NE) | 100 (NE, NE) |
| **12–16 years** |  |  |  |  |  |
| **Seropositive** | *n=1700/3402* | *n=1676/3355* | *n=1644/3284* | *n=1590/3179* | *n=1590/3179* |
| Case counts (placebo/TAK-003) | 15/2 | 2/1 | 7/3 | 1/0 | 1/0 |
| VE (95% CI) | 93.4 (71.0, 98.5) | 75.9 (–166.4, 97.8) | 79.9 (22.1, 94.8) | 100 (NE, NE) | 100 (NE, NE) |
| **Seronegative** | *n=324/669* | *n=321/659* | *n=316/648* | *n=305/626* | *n=305/626* |
| Case counts (placebo/TAK-003) | 2/0 | 1/0 | 1/1 | 1/0 | 1/0 |
| VE (95% CI) | 100 (NE, NE) | 100 (NE, NE) | 52.3 (–669.7, 97.0) | 100 (NE, NE) | 100 (NE, NE) |

Number of subjects evaluated each year may vary. Repeat episodes of VCD excluded from efficacy analysis.

Seronegative at baseline: seronegative to all four dengue serotypes. Seropositive at baseline: reciprocal neutralizing titer ≥10 for one or more dengue serotypes.
*30 days after 2^nd^ dose to end of 1^st^ year after 2^nd^ dose;

^†^This period generally includes Year 4 and the last 0.5 years were not uniform for all subjects.

CI, confidence interval; NE, non-estimable; VCD, virologically confirmed dengue; VE, vaccine efficacy

**Supplementary Table 6.** Clinical signs and symptoms of virologically-confirmed dengue (VCD) cases occurring from the first dose to the end of Part 3 (approximately Month 57 after the first dose) in 4–5 year-olds (safety set data). Includes data of second episodes of VCD (safety set).

|  | **Seropositive** | | **Seronegative** | |
| --- | --- | --- | --- | --- |
|  | **Placebo**  (n = 491) | **TAK-003**  (n = 1006) | **Placebo**  (n = 355) | **TAK-003**  (n = 696) |
| Number of VCD Cases | 63 | 59 | 33 | 49 |
| Duration of Febrile Illness  (Days; Median / Mean; 95% CI)* | 7.2 / 6.0 (6.0, 8.5) | 6.7 / 6.0 (5.7, 7.7) | 7.3 / 7.0 (6.3, 8.3) | 6.7 / 6.0 (5.5, 7.8) |
| Duration of Fever  (Days; Median / Mean; 95% CI) | 4.5 / 5.0 (4.0, 4.9) | 3.8 / 4.0 (3.5, 4.2) | 5.0 / 5.0 (4.3, 5.7) | 3.8 / 3.0 (3.3, 4.3) |
| Number (%) of  VCD Cases requiring Hospitalization | 8 (12.7) | 7 (11.9) | 7 (21.2) | 4 (8.2) |
| Duration of Hospitalization  (Days; Median / Mean; 95% CI) | 5.8 / 4.5 (3.6, 7.9) | 5.0 / 4.0 (3.3, 6.7) | 3.6 / 3.0 (2.8, 4.3) | 6.3 / 6.0 (3.0, 9.5) |
| Evidence of Bleeding  (%, n / N) | 9.5% (6/63) | 5.1% (3/59) | 6.1% (2/33) | 6.1% (3/49) |
| Plasma Leakage  (%, n / N) ^¶^ | 0% (0/63) | 0% (0/63) | 0% (0/33) | 4.1% (2/49) |
| Hematocrit Increase ≥ 20%  (%, n / N)^†^ | 16.7% (4/24) | 15.8% (3/19) | 7.1% (1/14) | 16.7% (2/12) |
| Platelet Count ≤ 100 x 10^9^ / L  (%, n / N)^‡^ | 5.7% (3/53) | 14.3% (6/42) | 8.7% (2/23) | 2/34 (5.9%) |
| Platelet Count ≤ 50 x 10^9^ / L  (%, n / N)^‡^ | 0% (0/53) | 4.8% (2/42) | 0% (0/23) | 0% (0/34) |
| ALT or AST ≥ 1000 U / L  (%, n / N)^‡^ | 0% (0/36) | 0% (0/22) | 0% (0/23) | 0% (0/34) |
| ALT or AST > 10x ULN (%, n / N) | 2.8% (1/36) | 0% (0/22) | 0% (0/23) | 0% (0/34) |
| Signs of Circulatory Failure (Any)  (%, n / N) | 0% (0/63) | 1.7% (1/59) | 0% (0/33) | 2.0% (1/49) |
| Reduced Pulse Pressure  (%, n / N) | 0% (0/63) | 1.7% (1/59) | 0% (0/33) | 0% (0/49) |
| Hypotensive Shock  (%, n / N) | 0% (0/63) | 0% (0/59) | 0% (0/33) | 2.0% (1/49) |

VCD, virologically-confirmed dengue; ALT, alanine aminotransferase; AST, aspartate aminotransferase; ULN, upper limit of normal; *duration of febrile illness defined as end date of latest symptom to start date of earliest symptom + 1 day (symptoms considered include fever and any general symptoms); ^¶^does not include hematocrit increase ≥ 20% reported in a separate row and is based on investigator reporting of clinical evidence of plasma leakage which could include clinical, radiological or laboratory findings; ^†^hematocrit increase defined as maximum hematocrit between Day 3 and Day 7 inclusive, from onset of fever ≥ 20% increase over minimum hematocrit before Day 3 or after Day 7 from onset of fever; ^‡^for platelet, ALT, and AST data, assessments within 14 days of onset of febrile illness have been considered (‘n’ in column header refers to number of participants in the Safety Set; ‘N’ in rows refers to number of VCD cases with available data for specific parameter)

**Supplementary Table 7.** Clinical signs and symptoms of virologically-confirmed dengue (VCD) cases occurring from the first dose to the end of Part 3 (approximately Month 57 after the first dose) in 6–11 year-olds (safety set data). Includes data of second episodes of VCD (safety set).

|  | **Seropositive** | | **Seronegative** | |
| --- | --- | --- | --- | --- |
|  | **Placebo**  (n = 2568) | **TAK-003**  (n = 5069) | **Placebo**  (n = 1128) | **TAK-003**  (n = 2315) |
| Number of VCD Cases | 299 | 165 | 98 | 84 |
| Duration of Febrile Illness  (Days; Median / Mean; 95% CI)* | 6.3 / 6.0 (5.9, 6.7) | 5.9 / 5.0 (5.4, 6.4) | 6.5 / 6.0 (5.6, 7.3) | 6.3 / 6.0 (5.7, 6.9) |
| Duration of Fever  (Days; Median / Mean; 95% CI) | 4.4 / 4.0 (4.2, 4.6) | 4.0 / 4.0 (3.8, 4.3) | 4.6 / 4.5 (4.3, 5.0) | 4.2 / 4.0 (3.9, 4.5) |
| Number (%) of  VCD Cases requiring Hospitalization | 60 (20.1) | 15 (9.1) | 29 (29.6) | 12 (14.3) |
| Duration of Hospitalization  (Days; Median / Mean; 95% CI) | 6.0 / 5.0 (4.1, 7.8) | 5.3 / 4.0 (2.4, 8.1) | 5.1 / 5.0 (4.4, 5.7) | 5.9 / 6.5 (4.9, 7.0) |
| Evidence of Bleeding  (%, n / N) | 7.9% (18/229) | 4.2% (7/165) | 5.1% (5/98) | 4.8% (4/84) |
| Plasma Leakage  (%, n / N) ^¶^ | 5.2% (12/229) | 0.6% (1/165) | 3.1% (3/98) | 4.8% (4/84) |
| Hematocrit Increase ≥ 20%  (%, n / N)^†^ | 16.1% (18/112) | 9.3% (4/43) | 5.7% (3/53) | 3.7% (1/27) |
| Platelet Count ≤ 100 x 10^9^ / L  (%, n / N)^‡^ | 28.5% (53/186) | 9.8% (10/102) | 15.6% (12/77) | 14.8% (8/54) |
| Platelet Count ≤ 50 x 10^9^ / L  (%, n / N)^‡^ | 12.4% (23/186) | 2.0% (2/102) | 6.5% (5/77) | 3.7% (2/54) |
| ALT or AST ≥ 1000 U / L  (%, n / N)^‡^ | 0.6% (1/155) | 0% (0/73) | 0% (0/68) | 0% (0/38) |
| ALT or AST > 10x ULN (%, n / N) | 1.9% (3/155) | 0% (0/73) | 1.5% (1/68) | 0% (0/38) |
| Signs of Circulatory Failure (Any)  (%, n / N) | 1.3% (3/229) | 0% (0/165) | 1.0% (1/98) | 0% (0/84) |
| Reduced Pulse Pressure  (%, n / N) | 0.9% (2/229) | 0% (0/165) | 0% (0/98) | 0% (0/84) |
| Hypotensive Shock  (%, n / N) | 0.4% (1/229) | 0% (0/165) | 1.0% (1/98) | 0% (0/84) |

VCD, virologically-confirmed dengue; ALT, alanine aminotransferase; AST, aspartate aminotransferase; ULN, upper limit of normal; *duration of febrile illness defined as end date of latest symptom to start date of earliest symptom + 1 day (symptoms considered include fever and any general symptoms); ^¶^does not include hematocrit increase ≥ 20% reported in a separate row and is based on investigator reporting of clinical evidence of plasma leakage which could include clinical, radiological or laboratory findings; ^†^hematocrit increase defined as maximum hematocrit between Day 3 and Day 7 inclusive, from onset of fever ≥ 20% increase over minimum hematocrit before Day 3 or after Day 7 from onset of fever; ^‡^for platelet, ALT, and AST data, assessments within 14 days of onset of febrile illness have been considered (‘n’ in column header refers to number of participants in the Safety Set; ‘N’ in rows refers to number of VCD cases with available data for specific parameter)

**Supplementary Table 8.** Clinical signs and symptoms of virologically-confirmed dengue (VCD) cases occurring from the first dose to the end of Part 3 (approximately Month 57 after the first dose) in 12–16 year-olds (safety set data). Includes data of second episodes of VCD (safety set).

|  | **Seropositive** | | **Seronegative** | |
| --- | --- | --- | --- | --- |
|  | **Placebo**  (n = 1795) | **TAK-003**  (n = 3588) | **Placebo**  (n = 349) | **TAK-003**  (n = 703) |
| Number of VCD Cases | 112 | 71 | 25 | 19 |
| Duration of Febrile Illness  (Days; Median / Mean; 95% CI)* | 6.7 / 6.0 (6.1, 7.2) | 6.2 / 6.0 (5.5, 6.9) | 7.7 / 7.0 (6.3, 9.1) | 6.4 / 7.0 (5.4, 7.4) |
| Duration of Fever  (Days; Median / Mean; 95% CI) | 4.7 / 5.0 (4.3, 5.0) | 4.2 / 4.0 (3.8, 4.6) | 5.0 / 5.0 (4.3, 5.7) | 3.9 / 4.0 (3.3, 4.6) |
| Number (%) of  VCD Cases requiring Hospitalization | 33 (29.5) | 7 (9.9) | 5 (20.0) | 1 (5.3) |
| Duration of Hospitalization  (Days; Median / Mean; 95% CI) | 5.1 / 5.0 (4.3, 5.9) | 4.0 / 3.0 (2.4, 5.6) | 5.4 / 5.0 (3.1, 7.7) | 5.0 / 5.0 (NE, NE) |
| Evidence of Bleeding  (%, n / N) | 8.0% (9/112) | 4.2% (3/71) | 4.0% (1/25) | 5.3% (1/19) |
| Plasma Leakage  (%, n / N) ^¶^ | 5.4% (6/112) | 1.4% (1/71) | 4.0% (1/25) | 5.3% (1/19) |
| Hematocrit Increase ≥ 20%  (%, n / N)^†^ | 12.0% (6/50) | 4.0% (1/25) | 0% (0/11) | 33.3% (1/3) |
| Platelet Count ≤ 100 x 10^9^ / L  (%, n / N)^‡^ | 31.8% (27/85) | 11.8% (6/51) | 18.8% (3/16) | 12.5% (1/8) |
| Platelet Count ≤ 50 x 10^9^ / L  (%, n / N)^‡^ | 15.3% (13/85) | 5.9% (3/51) | 0% (0/16) | 12.5% (1/8) |
| ALT or AST ≥ 1000 U / L  (%, n / N)^‡^ | 0% (0/69) | 0% (0/33) | 0% (0/14) | 0% (0/7) |
| ALT or AST > 10x ULN (%, n / N) | 4.3% (3/69) | 0% (0/33) | 0% (0/14) | 0% (0/7) |
| Signs of Circulatory Failure (Any)  (%, n / N) | 0% (0/112) | 0% (0/71) | 0% (0/25) | 0% (0/19) |
| Reduced Pulse Pressure  (%, n / N) | 0% (0/112) | 0% (0/71) | 0% (0/25) | 0% (0/19) |
| Hypotensive Shock  (%, n / N) | 0% (0/112) | 0% (0/71) | 0% (0/25) | 0% (0/19) |

VCD, virologically-confirmed dengue; ALT, alanine aminotransferase; AST, aspartate aminotransferase; ULN, upper limit of normal; NE, not evaluable *duration of febrile illness defined as end date of latest symptom to start date of earliest symptom + 1 day (symptoms considered include fever and any general symptoms); ^¶^does not include hematocrit increase ≥ 20% reported in a separate row and is based on investigator reporting of clinical evidence of plasma leakage which could include clinical, radiological or laboratory findings; ^†^hematocrit increase defined as maximum hematocrit between Day 3 and Day 7 inclusive, from onset of fever ≥ 20% increase over minimum hematocrit before Day 3 or after Day 7 from onset of fever; ^‡^for platelet, ALT, and AST data, assessments within 14 days of onset of febrile illness have been considered (‘n’ in column header refers to number of participants in the Safety Set; ‘N’ in rows refers to number of VCD cases with available data for specific parameter)

**Supplementary Table 9.** Distribution of severe and DHF cases and causative serotype by age-group from first vaccination to the end of Part 3 (safety set)

|  | **Baseline Seropositive** | | **Baseline Seronegative** | |
| --- | --- | --- | --- | --- |
| **Age-group** | **Placebo** | **TAK-003** | **Placebo** | **TAK-003** |
| **Severe dengue** |  |  |  |  |
| 4–5 years | *n=491* | *n=1006* | *n=355* | *n=696* |
|  | 1  (DENV-3) | 1*  (DENV-3) | 0 | 1*  (DENV-3) |
| 6–11 years | *n=2568* | *n=5069* | *n=1128* | *n=2315* |
|  | 4*  (DENV-1 n=1; DENV-2 n=1; DENV-3 n=2) | 0 | 0 | 1  (DENV-3) |
| 12–16 years | *n=1795* | *n=3588* | *n=349* | *n=703* |
|  | 0 | 0 | 0 | 0 |
| **DHF** |  |  |  |  |
| 4–5 years | *n=491* | *n=1006* | *n=355* | *n=696* |
|  | 0 | 2*  (DENV-1 n=1; DENV-3 n=1) | 0 | 2*  (DENV-3) |
| 6–11 years | *n=2568* | *n=5069* | *n=1128* | *n=2315* |
|  | 7*  (DENV-1 n=2; DENV-2 n=3; DENV-3 n=2) | 1  (DENV-1) | 1  (DENV-1) | 1  (DENV-3) |
| 12–16 years | *n=1795* | *n=3588* | *n=349* | *n=703* |
|  | 6  (DENV-1 n=1; DENV-2 n=4; DENV-4 n=1) | 2  (DENV-3 n=2) | 1  (DENV-3) | 1  (DENV-3) |

DHF, dengue hemorrhagic fever

*one participant in the group met criteria for both severe dengue and DHF and is included in both categories

**Supplementary Table 10.** Summary of solicited local AEs up to 7 days and systemic AEs up to 14 days after any vaccination by age group and baseline serostatus (safety set immunogenicity subset)

|  | **Seropositive** | | **Seronegative** | |
| --- | --- | --- | --- | --- |
|  | **Placebo** | **TAK-003** | **Placebo** | **TAK-003** |
| **4–5 years** |  |  |  |  |
| Local AEs | 20/97 (20.6) | 58/197 (29.4) | 23/72 (31.9) | 48/134 (35.8) |
| Systemic AEs | 30/97 (30.9) | 69/197 (35.0) | 21/72 (29.2) | 40/134 (29.9) |
| Related systemic AEs | 20/97 (20.6) | 50/197 (25.4) | 13/72 (18.1) | 24/134 (17.9) |
|  |  |  |  |  |
| **6–11 years** |  |  |  |  |
| Local AEs | 136/510 (26.7) | 365/1002 (36.4) | 61/215 (28.4) | 181/454 (39.9) |
| Systemic AEs | 200/510 (39.2) | 412/1003 (41.1) | 78/215 (36.3) | 182/454 (40.1) |
| Related systemic AEs | 151/510 (29.6) | 298/1003 (29.7) | 55/215 (25.6) | 140/454 (30.8) |
|  |  |  |  |  |
| **12–16 years** |  |  |  |  |
| Local AEs | 78/343 (22.7) | 251/706 (35.6) | 20/80 (25.0) | 64/140 (45.7) |
| Systemic AEs | 135/343 (39.4) | 327/707 (46.3) | 36/80 (45.0) | 75/140 (53.6) |
| Related systemic AEs | 100/343 (29.2) | 248/707 (35.1) | 31/80 (38.8) | 60/140 (42.9) |

Data are for n/N (%) unless otherwise stated. Solicited local AEs were considered related by default.

**Supplementary Table 11**. Summary of unsolicited AEs up to 28 days after any vaccination by age group and baseline serostatus (safety set immunogenicity subset)

|  | **Seropositive** | | **Seronegative** | |
| --- | --- | --- | --- | --- |
|  | **Placebo** | **TAK-003** | **Placebo** | **TAK-003** |
| **4–5 years** | *n=97* | *n=200* | *n=72* | *n=141* |
| Any AE | 29 (29.9) | 54 (27.0) | 13 (18.1) | 34 (24.1) |
| Mild | 24 (24.7) | 49 (24.5) | 13 (18.1) | 30 (21.3) |
| Moderate | 5 (5.2) | 5 (2.5) | 0 (0.0) | 4 (2.8) |
| Severe | 0 (0.0) | 0 (0.0) | 0 (0.0) | 0 (0.0) |
| Related AE | 3 (3.1) | 4 (2.0) | 0 (0.0) | 1 (0.7) |
| Leading to discontinuation | 0 (0.0) | 0 (0.0) | 0 (0.0) | 0 (0.0) |
|  |  |  |  |  |
| **6–11 years** | *n=516* | *n=1013* | *n=217* | *n=456* |
| Any AE | 91 (17.6) | 180 (17.8) | 39 (18.0) | 73 (16.0) |
| Mild | 77 (14.9) | 138 (13.6) | 31 (14.3) | 54 (11.8) |
| Moderate | 14 (2.7) | 39 (3.8) | 8 (3.7) | 15 (3.3) |
| Severe | 0 (0.0) | 3 (0.3) | 0 (0.0) | 4 (0.9) |
| Related AE | 2 (0.4) | 7 (0.7) | 3 (1.4) | 4 (0.9) |
| Leading to discontinuation | 0 (0.0) | 1 (<0.1) | 0 (0.0) | 0 (0.0) |
|  |  |  |  |  |
| **12–16 years** | *n=347* | *n=710* | *n=80* | *n=143* |
| Any AE | 63/347 (18.2) | 121 (17.0) | 14 (17.5) | 25 (17.5) |
| Mild | 52 (15.0) | 97 (13.7) | 11 (13.8) | 23 (16.1) |
| Moderate | 11 (3.2) | 21 (3.0) | 3 (3.8) | 2 (1.4) |
| Severe | 0 (0.0) | 3 (0.4) | 0 (0.0) | 0 (0.0) |
| Related AE | 10 (2.9) | 6 (0.8) | 0 (0.0) | 1 (0.7) |
| Leading to discontinuation | 0 (0.0) | 1 (0.1) | 0 (0.0) | 1 (0.7) |

Data are for n (%) unless otherwise stated.

**Supplementary Table 12.** Most frequently reported SAEs (>0.2% participants in any group) by age group during Parts 1 and 2 of the study (safety set). Data are presented for number (%) of participants

| **MedDRA System Organ Class/Preferred Term** | **Placebo** | **TAK-003** |
| --- | --- | --- |
| **4–5 years** | *N=846* | *N=1702* |
| Gastrointestinal disorders | 3 (0.4) | 2 (0.1) |
| Infections and infestations | 27 (3.2) | 62 (3.6) |
| Gastroenteritis | 3 (0.4) | 10 (0.6) |
| Dengue fever | 6 (0.7) | 5 (0.3) |
| Pneumonia | 5 (0.6) | 5 (0.3) |
| Influenza | 2 (0.2) | 6 (0.4) |
| Lower respiratory tract infection | 2 (0.2) | 6 (0.4) |
| Viral infection | 3 (0.4) | 4 (0.2) |
| Injury, poisoning and procedural complications | 4 (0.5) | 17 (1.0) |
| Nervous system disorders | 2 (0.2) | 6 (0.4) |
| Respiratory, thoracic and mediastinal disorders | 2 (0.2) | 6 (0.4) |
| Asthma | 2 (0.2) | 6 (0.4) |
| **6–11 years** | *N=3697* | N=7387 |
| Gastrointestinal disorders | 7 (0.2) | 20 (0.3) |
| Infections and infestations | 143 (3.9) | 172 (2.3) |
| Dengue fever | 40 (1.1) | 13 (0.2) |
| Appendicitis | 9 (0.2) | 23 (0.3) |
| Gastroenteritis | 5 (0.1) | 22 (0.3) |
| Viral infection | 11 (0.3) | 15 (0.2) |
| Dengue haemorrhagic fever | 18 (0.5) | 1 (<0.1) |
| Pneumonia | 10 (0.3) | 8 (0.1) |
| Injury, poisoning and procedural complications | 28 (0.8) | 61 (0.8) |
| **12–16 years** | *N=2144* | N=4291 |
| Infections and infestations | 57 (2.7) | 75 (1.7) |
| Dengue fever | 16 (0.7) | 4 (<0.1) |
| Appendicitis | 10 (0.5) | 20 (0.5) |
| Viral infection | 9 (0.4) | 7 (0.2) |
| Dengue haemorrhagic fever | 7 (0.3) | 1 (<0.1) |
| Injury, poisoning and procedural complications | 17 (0.8) | 33 (0.8) |
| Pregnancy, puerperium and perinatal conditions | 4 (0.2) | 12 (0.3) |

**Supplementary Table 13.** Most frequently reported SAEs (>0.2% participants in any group) by age group during Part 3 of the study (safety set). Data are presented for number (%) of participants

| **MedDRA System Organ Class/Preferred Term** | **Placebo** | **TAK-003** |
| --- | --- | --- |
| **4–5 years** | *N=846* | *N=1702* |
| Gastrointestinal disorders | 3 (0.4) | 5 (0.3) |
| Infections and infestations | 32 (3.8) | 54 (3.2) |
| Dengue fever | 10 (1.2) | 11 (0.6) |
| Appendicitis | 2 (0.2) | 6 (0.4) |
| Viral infection | 3 (0.4) | 4 (0.2) |
| Injury, poisoning and procedural complications | 4 (0.5) | 18 (1.1) |
| Animal bite | 1 (0.1) | 7 (0.4) |
| Nervous system disorders | 3 (0.4) | 3 (0.2) |
| **6–11 years** | *N=3697* | N=7387 |
| Gastrointestinal disorders | 13 (0.4) | 15 (0.2) |
| Infections and infestations | 155 (4.2) | 234 (3.2) |
| Dengue fever | 52 (1.4) | 34 (0.5) |
| Appendicitis | 14 (0.4) | 29 (0.4) |
| COVID-19 | 3 (<0.1) | 19 (0.3) |
| Viral infection | 12 (0.3) | 7 (<0.1) |
| Injury, poisoning and procedural complications | 18 (0.5) | 51 (0.7) |
| **12–16 years** | *N=2144* | N=4291 |
| Gastrointestinal disorders | 5 (0.2) | 11 (0.3) |
| Infections and infestations | 83 (3.9) | 133 (3.1) |
| Dengue fever | 21 (1.0) | 11 (0.3) |
| Appendicitis | 11 (0.5) | 25 (0.6) |
| Asymptomatic COVID-19 | 5 (0.2) | 17 (0.4) |
| COVID-19 | 6 (0.3) | 12 (0.3) |
| Urinary tract infection | 9 (0.4) | 6 (0.1) |
| Injury, poisoning and procedural complications | 25 (1.2) | 34 (0.8) |
| Pregnancy, puerperium and perinatal conditions | 21 (1.0) | 38 (0.9) |
| Abortion spontaneous | 7 (0.3) | 5 (0.1) |
| Psychiatric disorders | 3 (0.1) | 16 (0.4) |

**Supplementary Figure 1.** Seropositivity rates throughout the study by treatment group and baseline serostatus for participants aged (a) 4–5 years, (b) 6–11 years, and (c) 12–16 years at baseline (per protocol set for immunogenicity). Note that number of participants included at individual time points may vary

(a)


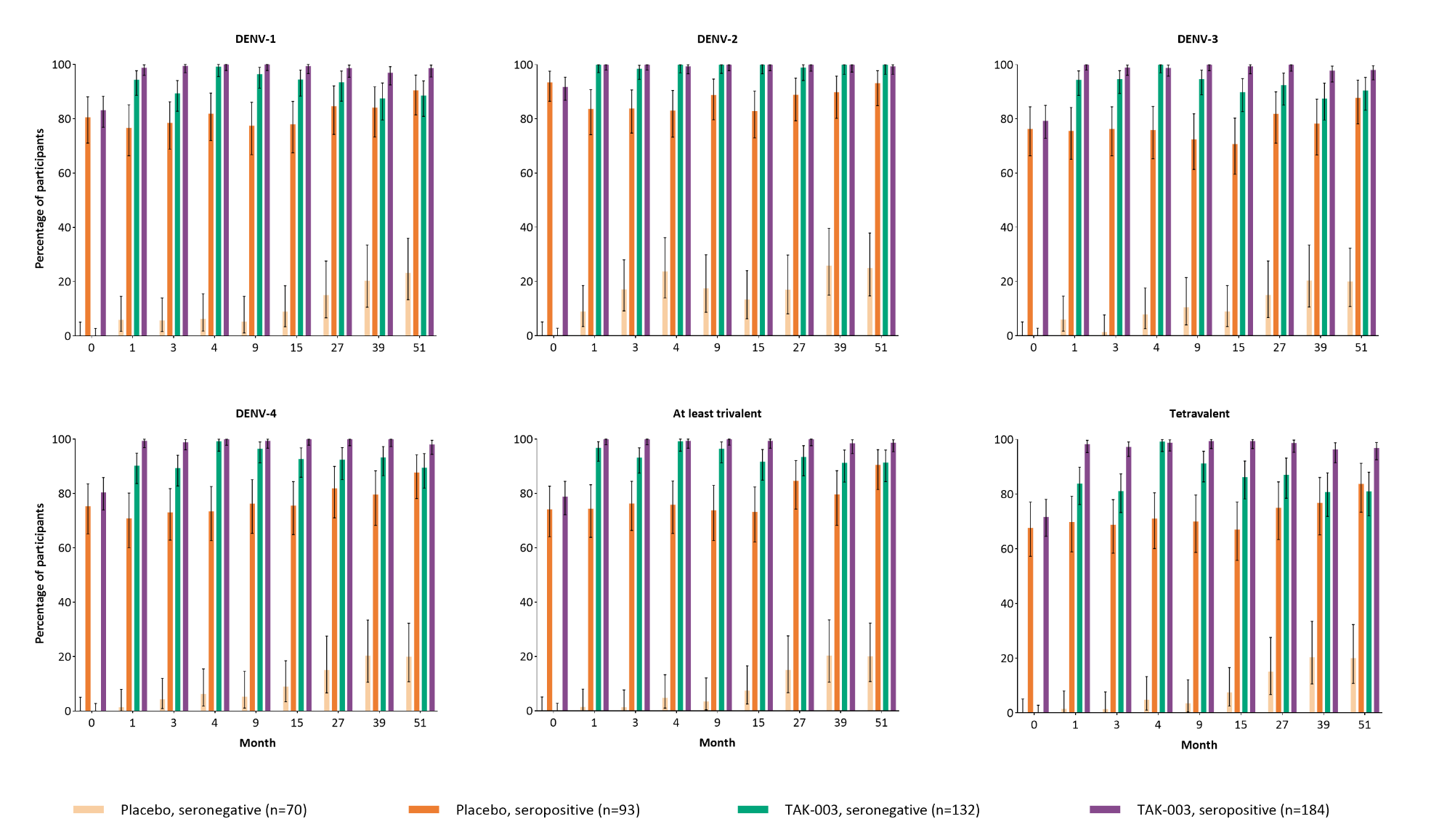


(b)


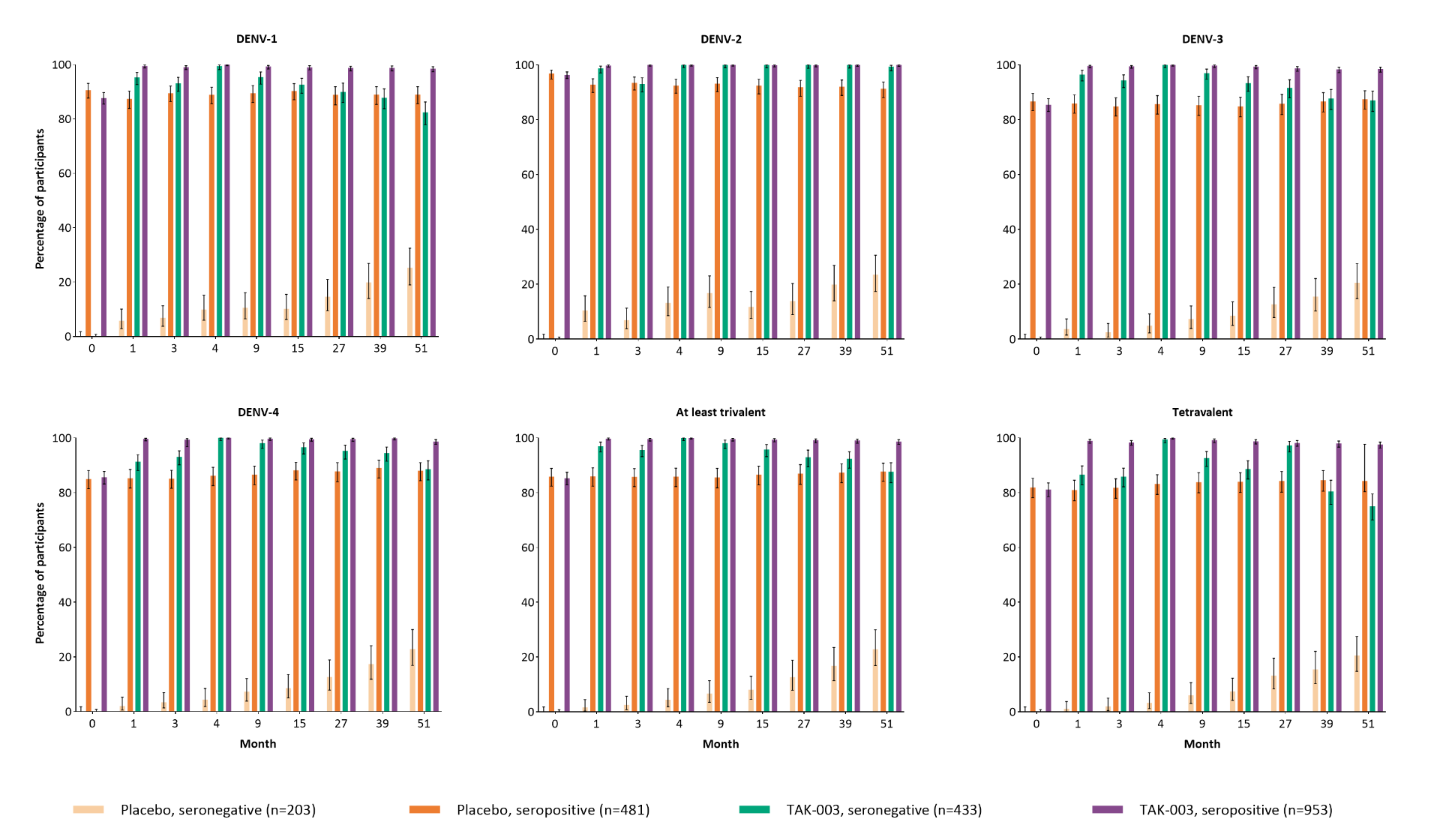


(c)


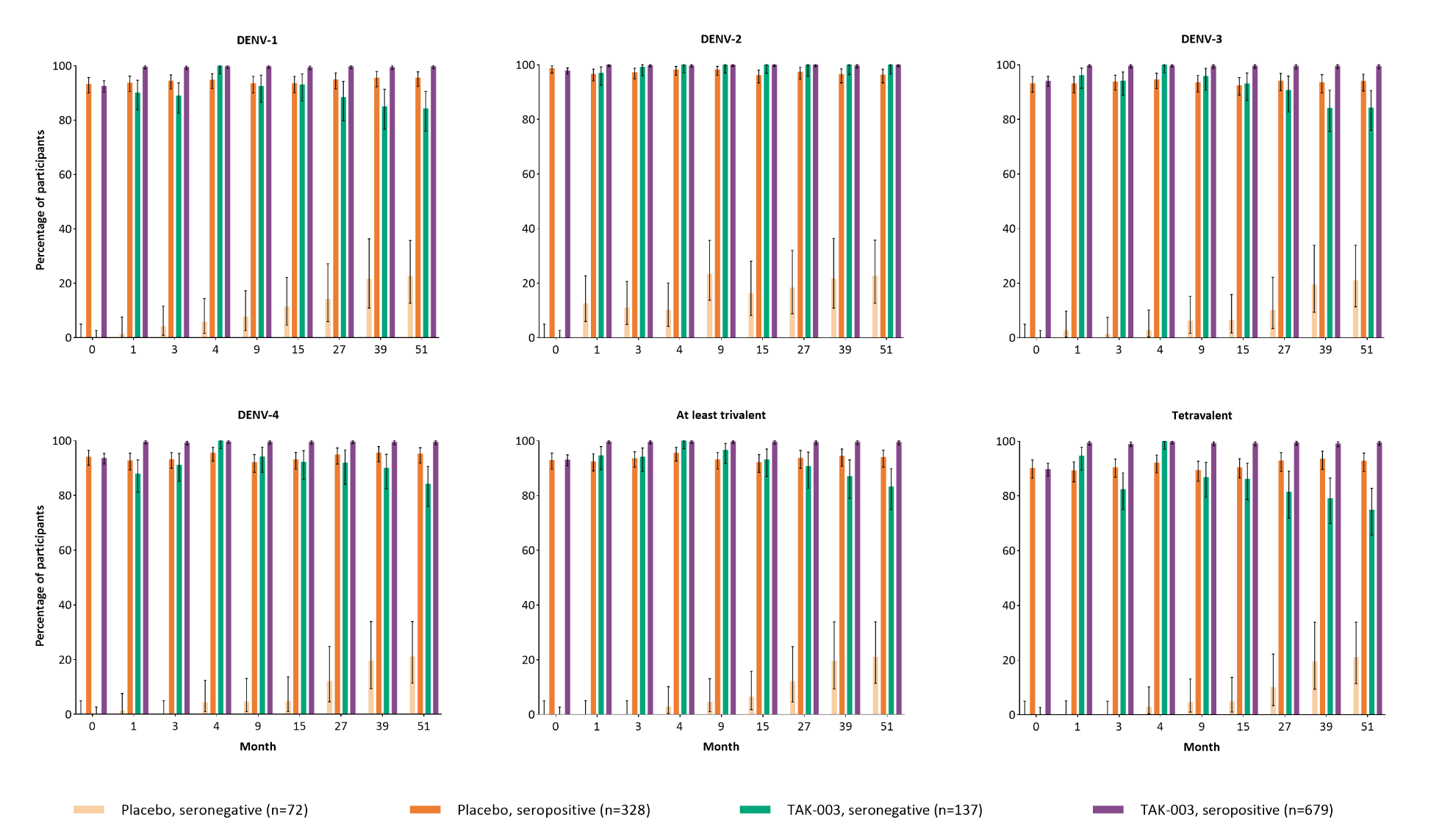

Supplement: ciae369_Supplementary_Data [file ciae369_supplementary_data.docx]
